# Suppression of mitochondrial ROS by prohibitin drives glioblastoma progression and therapeutic resistance

**DOI:** 10.1038/s41467-021-24108-6

**Published:** 2021-06-17

**Authors:** Haohao Huang, Songyang Zhang, Yuanyuan Li, Zhaodan Liu, Lanjuan Mi, Yan Cai, Xinzheng Wang, Lishu Chen, Haowen Ran, Dake Xiao, Fangye Li, Jiaqi Wu, Tingting Li, Qiuying Han, Liang Chen, Xin Pan, Huiyan Li, Tao Li, Kun He, Ailing Li, Xuemin Zhang, Tao Zhou, Qing Xia, Jianghong Man

**Affiliations:** 1 State Key Laboratory of Proteomics, National Center of Biomedical Analysis, Beijing, China; 2 Nanhu Laboratory, Jiaxing, Zhejiang Province, China; 3 Department of Neurosurgery, General Hospital of Central Theater Command of Chinese People’s Liberation Army, Wuhan, PR China; 4 Department of Neurosurgery, First Medical Center of PLA General Hospital, Beijing, China; 5 The First Hospital of Jilin University, Changchun, China; 6 State Key Laboratory of Toxicology and Medical Countermeasures, Beijing Institute of Pharmacology and Toxicology, National Center of Biomedical Analysis, Beijing, China

**Keywords:** Cancer stem cells, CNS cancer

## Abstract

Low levels of reactive oxygen species (ROS) are crucial for maintaining cancer stem cells (CSCs) and their ability to resist therapy, but the ROS regulatory mechanisms in CSCs remains to be explored. Here, we discover that prohibitin (PHB) specifically regulates mitochondrial ROS production in glioma stem-like cells (GSCs) and facilitates GSC radiotherapeutic resistance. We find that PHB is upregulated in GSCs and is associated with malignant gliomas progression and poor prognosis. PHB binds to peroxiredoxin3 (PRDX3), a mitochondrion-specific peroxidase, and stabilizes PRDX3 protein through the ubiquitin-proteasome pathway. Knockout of *PHB* dramatically elevates ROS levels, thereby inhibiting GSC self-renewal. Importantly, deletion or pharmacological inhibition of PHB potently slows tumor growth and sensitizes tumors to radiotherapy, thus providing significant survival benefits in GSC-derived orthotopic tumors and glioblastoma patient-derived xenografts. These results reveal a selective role of PHB in mitochondrial ROS regulation in GSCs and suggest that targeting PHB improves radiotherapeutic efficacy in glioblastoma.

## Abstract (web summary)

How ROS levels are regulated in cancer stem cells and their contribution to cancer resistance is currently not clear. Here, the authors show that prohibitin regulates mitochondrial ROS production stabilizing the peroxidase PRDX3 and this accounts for radiotherapy resistance in glioma stem-like cells.

## Introduction

Glioblastoma (GBM) is the most common and lethal type of human primary brain tumor with an extremely poor prognosis^1–3^. Ionizing radiation (IR) is one of the standard nonsurgical treatments for nearly all GBM^4,5^. Unfortunately, the efficacy of radiotherapy in GBM is dismal partially due to the radioresistance of cancer stem cells (CSCs) in GBM, termed glioma stem-like cells (GSCs)^6–10^. CSCs are functionally defined by their capacity to maintain tumor heterogeneity, drive tumor growth, and therapy resistance^9,11^. Currently, there is no effective targeted therapy for CSCs in GBM treatment. Thus, the identification of essential GSC-specific regulators may provide insights into novel therapeutic strategies against GSCs.

Reactive oxygen species (ROS) are important byproducts of cell metabolism. The maintenance of ROS homeostasis is crucial for cancer cell survival^12–14^. Similar to normal tissue stem cells, CSCs may contain lower levels of ROS relative to bulk tumor cells (BTCs), and this promotes CSC self-renewal and tumor therapeutic resistance^15^. This raises a critical question of whether CSCs and normal stem cells share the same mechanisms to meet redox stress, a process that poses a major challenge for targeting ROS as a radical therapeutic approach for CSCs. However, the regulatory mechanisms of ROS in CSCs are not fully understood.

To adapt to oxidative stress, cancer cells develop a ROS-scavenging system to eliminate the over-accumulation of ROS^16–19^. Peroxiredoxins (PRDXs) are a family of thiol peroxidases that scavenge peroxides in cells. Mammalian cells possess six PRDX isoforms, with PRDX1, 2, and 6 in the cytoplasm, PRDX3 in the mitochondria, PRDX4 in the endoplasmic reticulum, and PRDX5 distributed throughout of the cell^20^. The PRDX family proteins are divided into three classes (2-Cys, atypical 2-Cys, and 1-Cys) based on the number of Cys residues that participate in catalysis. PRDX3 belongs to the 2-Cys subgroup and exists as a homodimer. In the catalytic cycle of PRDXs, H_2_O_2_ oxidizes the catalytic thiol (Cys-SH) to sulfenic acid (Cys-SOH), and this then reacts with the second conserved Cys-SH of the other subunit in the homodimer to form an intersubunit disulfide. This disulfide is subsequently reduced by thioredoxin (Trx) to complete the catalytic cycle^21,22^. Mitochondria are a major source of ROS. Kinetic analysis indicates that PRDX3 contributes to the majority of hydrogen peroxide reduction in mitochondria^19,22^, suggesting a crucial role for PRDX3 in the maintenance of mitochondrial redox balance. Although members of the PRDX family have been linked to malignant progression and chemotherapeutic resistance in certain types of tumors^20,23^, the role of PRDX3 in CSCs remains unknown.

Prohibitin (PHB) and its homolog PHB2, comprising the PHB family of proteins, are highly conserved pleiotropic proteins with multiple functions in different cell types^24–26^. PHB was originally identified as a tumor suppressor, as it co-localizes with p53 and pRb in the nucleus of breast cancer cells and promotes cell apoptosis^27,28^. It has also been suggested that PHB is localized in the plasma membrane where it activates PI3K/Akt and C-Raf/ERK signaling pathways in cervical carcinoma cell lines and T cells^29,30^. Recently, a study has shown that PHB forms a complex with HIRA in the nucleus of human embryonic stem cells to regulate chromatin organization, suggesting an unexpected role of PHB in stem cell maintenance^31^. While the function of PHB is controversial, most studies have provided insights into the roles of PHB in mitochondrial biology^25^. The diverse functions of PHB may be modulated in a tissue-dependent manner, thus indicating that targeting PHB could provide a useful strategy for cancer treatment. In this study, we identified PHB as a specific regulator of ROS in GSCs. PHB is highly expressed in GSCs relative to non-stem tumor cells (NSTCs), neural progenitor cells (NPCs), and normal brain cells. PHB binds to and stabilizes PRDX3, to thereby inhibit mitochondrial ROS accumulation and promote GSC self-renewal and radioresistance. Targeting GSCs by genetic deletion or pharmacological inhibition of PHB potently inhibits GBM growth and overcomes the resistance of GBM to radiation treatment, highlighting that PHB blockade synergizes with radiotherapy in GBM treatment.

## Results

### PHB is highly expressed in GSCs

Our previous mass spectrometry screening identified a series of differentially expressed proteins between GSCs and their matched differentiated NSTCs^32^. Among them, 35 proteins were upregulated in four GSC lines relative to NSTCs (average fold change >2) (Supplementary Fig. 1a). To select specific candidates for further study, we used the following criteria: (1) the candidate should be abundantly expressed in GSCs; (2) the candidate should be associated with malignant progression of glioma; and (3) the candidate could be targeted by small molecular inhibitors. After candidate filtering with these criteria, we focused on PHB, an evolutionarily conserved and multifunctional protein^24,25^, whose role in GSCs remains unknown.

**Fig. 1:**
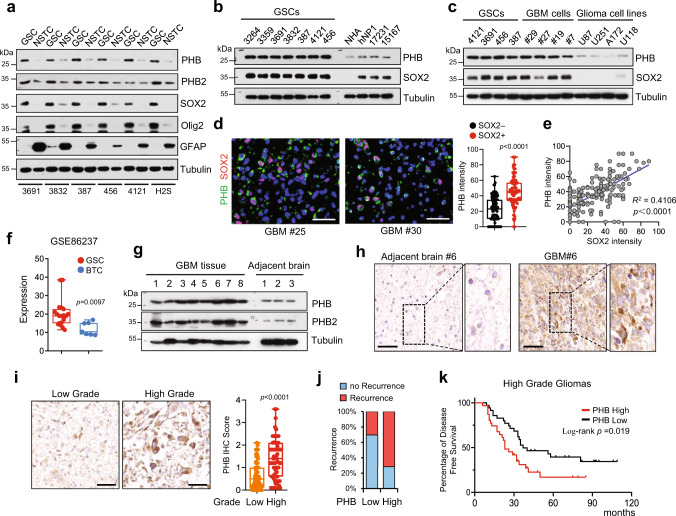
PHB is highly expressed in GSCs. **a**–**c** Immunoblot (IB) showing the expression of indicated proteins in glioma stem-like cells (GSCs) and matched non-stem tumor cells (NSTCs) (**a**), in GSCs, normal human astrocyte (NHA), and human neural progenitor cells (hNP1, 17231, and 15167) (**b**), or in GSCs, primary GBM cells and established glioma cell lines (**c**). **d** and **e** Representative immunofluorescent (IF) images of human primary glioblastoma (GBM) specimens stained with anti-PHB (green) and anti-SOX2 (red). Nuclei were counterstained with Hoechst (blue) (**d**, left). Scale bars, 40 μm. Quantifications of PHB staining intensity in SOX2+ (*n* = 110) and SOX2− (*n* = 100) cells (five random microscope fields from three tumors) are shown (**d**, right). Pearson’s correlation coefficient between PHB and SOX2 staining intensity in GBM cells is shown (**e**). **f** mRNA expression of PHB in GSCs (*n* = 19) relative to bulk tumor cells (BTCs, *n* = 7) from GEO profile (GSE86237) are shown. **g** IB showing the levels of PHB and PHB2 in human primary GBM tissues and adjacent normal brain tissues. **h** Immunohistochemical (IHC) staining of PHB in primary GBM and matched adjacent brain tissue. Scale bars, 100 μm. **i**–**k** IHC analysis of PHB in a glioma tissue microarray. Representative images and boxplots of histoscore of PHB in low grade and high grade gliomas are shown (**i**). Scale bars, 50 μm. (low grade, *n* = 94; high grade, *n* = 66). The percentages of recurrence of gliomas in tumors with low (*n* = 79) or high (*n* = 81) expression of PHB are shown (**j**). Kaplan–Meier survival analysis of patients with PHB low (*n* = 35) and PHB high (*n* = 31) expression in high-grade gliomas are shown (**k**). (Log-rank Mantel–Cox test). See Supplementary Table 1. Boxplots represent the median, 25th, and 75th percentiles. The maximum and minimum are connected to the center box through the vertical lines (whiskers). **d**, **f**, **i** Unpaired two-sided Student’s *t*-test (**d**, **f**), Welch’s two-sided *t-*test (**i**). Source data are provided as Source Data file.

To confirm the upregulation of PHB in GSCs, we first performed immunoblot (IB) analysis in multiple GSCs that were functionally characterized in studies including ours^32–35^. The results showed that PHB was highly expressed in all six GSC lines relative to the matched NSTCs (Fig. 1a and Supplementary Fig. 1b). PHB2, a homolog of PHB, was only increased in three of the six GSC lines compared with NSTCs (Fig. 1a, 3691, 3832, and H2S). Moreover, PHB levels were gradually decreased during GSC differentiation induced by serum (Supplementary Fig. 1c). Additionally, PHB expression was dramatically elevated in GSCs and tumor cells isolated from GBM specimens, compared to levels in normal human astrocytes (NHA), NPCs, and established glioma cell lines (Fig. 1b, c). To assess the expression of PHB in GSCs in vivo, we performed co-immunofluorescence (Co-IF) staining of PHB and the stem cell markers SOX2 or Olig2 in primary GBM specimens. The results showed that SOX2+ or Olig2+ cells were enriched in cells with high PHB staining (Fig. 1d and Supplementary Fig. 1d). PHB levels were positively correlated with the expression of SOX2 and Olig2 in GBM tissues (Fig. 1e and Supplementary Fig. 1e). We further analyzed two independent Gene Expression Omnibus (GEO) databases (GSE86237 and GSE54791)^36,37^, and we found that *PHB* was significantly increased in GSCs relative to BTCs (Fig. 1f) or the matched differentiated glioma cells (DGCs) (Supplementary Fig. 1f), supporting the upregulation of PHB in GSCs.

To investigate the clinical relevance of PHB in glioma, we performed IB analysis using fresh GBM specimens. We found that the expression of PHB was strongly increased in GBM tissues relative to adjacent normal brain tissues (Fig. 1g). Immunohistochemical (IHC) staining showed that PHB was strongly expressed in a subpopulation of tumor cells in GBM tissues but was barely expressed in normal brain (Fig. 1h and Supplementary Fig. 1g). Moreover, IHC staining using a glioma tissue microarray demonstrated that both the percentage of PHB+ cells and the intensity of PHB staining were significantly higher in high-grade gliomas relative to low-grade gliomas (Fig. 1i and Supplementary Table 1). Importantly, glioma patients with high PHB IHC scores exhibited increased recurrence (Fig. 1j and Supplementary Fig. 1h). In high-grade gliomas (grades III and IV), patients with high PHB IHC scores were associated with poor survival (Fig. 1k).

*PHB* possesses a highly conserved 3′-untranslated region (UTR) containing a putative microRNA-27a biding site^38^ (Supplementary Fig. 2a), suggesting that miR-27a may regulate the expression of *PHB* in GSCs. To test this possibility, we first examined the levels of miR-27a in GSCs, matched NSTCs and normal brain cells. In contrast to the high expression of PHB in GSCs, miR-27a was highly expressed in NSTCs, NHA, and hNP1 (Supplementary Fig. 2b, c). Transduction of GSCs with miR-27a mimics significantly decreased the expression of *PHB* (Supplementary Fig. 2d, left). However, transduction of the miR-27a inhibitor upregulated the mRNA levels of *PHB* in GSCs (Supplementary Fig. 2d, right). To further explore whether miR-27a suppresses *PHB* expression directly through the putative binding site in the 3′UTR of *PHB*, we generated luciferase reporter constructs containing the wild-type or mutated miR-27a-binding site (Supplementary Fig. 2e). Co-transfection of the wild-type binding site reporter with miR-27a mimic or with miR-27a inhibitor, respectively, decreased or increased luciferase reporter activity (Supplementary Fig. 2f). However, the activity of luciferase reporter with a mutated *PHB* 3′UTR was not significantly altered by the miR-27a mimic (Supplementary Fig. 2g). Additionally, the repression of miR-27a on PHB expression was validated at the protein level in GSCs (Supplementary Fig. 2h). These data suggest that miR-27a mediates the high expression of PHB in GSCs.

Taken together, these results demonstrate that PHB is highly expressed in GSCs and associated with the malignant progression of gliomas.

### PHB promotes GSC self-renewal and tumor progression

**Fig. 2:**
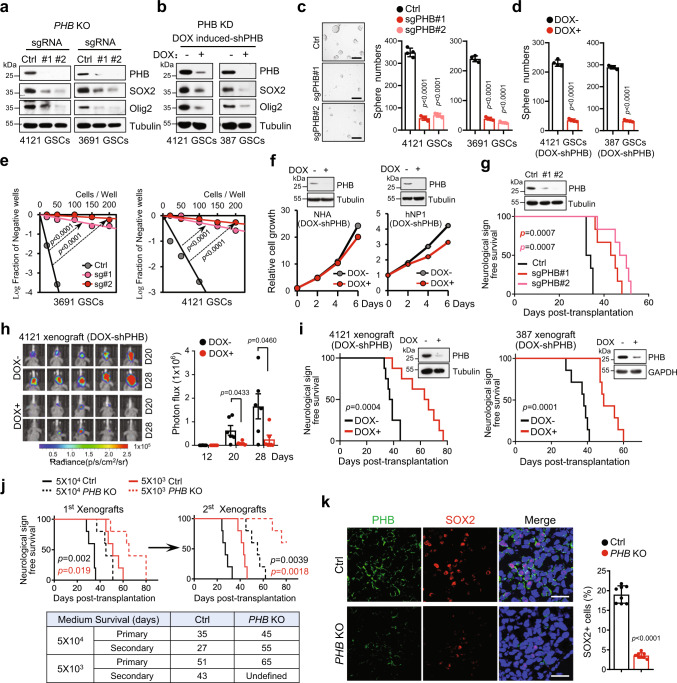
PHB promotes GSC self-renewal and tumor progression. **a** *PHB* knockout GSCs were generated by CRISPR-Cas9 system. IB showing levels of indicated proteins in Ctrl and *PHB* KO GSCs. **b** GSCs stably transduced with Tet-on-inducible-shPHB were treated with DOX (100 ng/ml) or vehicle control. Expression of indicated proteins were assessed by IB. **c**, **d** Quantifications of tumorsphere numbers (2000 cells/well) formed by Ctrl or *PHB* KO GSCs (**c**, right), or control or PHB inducible-KD GSCs (**d**) (mean ± SD, *n* = 4, biologically independent experiments). Representative images of tumorspheres are shown (**c**, left). Scale bar, 100 μm. **e** In vitro extreme limiting dilution assays (ELDAs) show that *PHB* KO decreased the frequency of tumorsphere formation in GSCs. **f** Inducible-KD of PHB had limit effects on cell growth of NHA (left) and hNP1 (right), as measured by cell viability assay. **g`** Ctrl or *PHB* KO 4121 GSCs (5 × 10^4^/mouse) were implanted into the brains of nude mice (nu/nu, *n* = 6). Kaplan–Meier survival curve of mice is shown (Log-rank Mantel–Cox test). IB showing the efficiency of *PHB* KO in xenografts (top). **h**, **i** GSCs transduced with Tet-on-inducible-shPHB and Luciferase reporter were implanted into the brains of nude mice (nu/nu). Mice were treated with vehicle control or DOX (2 mg/ml in drinking water) to induce expression of shPHB from day 0. GBM xenografts (4121 GSCs) were tracked by bioluminescence (**h**, left). Bioluminescent quantification of tumor growth is shown (**h**, right) (mean ± SEM, *n* = 6, biologically independent mice). Kaplan–Meier survival curves of mice are shown (**i**) (4121 GSCs, *n* = 8; 387 GSCs, *n* = 7; Log-rank Mantel-Cox test). IB showing the efficiency of PHB knockdown in the xenografts (top). **j** The in vivo serial transplantation assay shows that *PHB* KO inhibits GSC self-renewal in vivo. Kaplan–Meier survival curves of mice implanted with indicated GSCs (4121) are shown (top) (*n* = 5; Log-rank Mantel–Cox test). Summary of mice medium survival in the serial transplantation assay is shown (bottom). **k** Co-IF staining of PHB (green) and SOX2 (red) in GBM xenografts derived from Ctrl or *PHB* KO GSCs (4121). Quantifications of SOX2+ cells are shown (right) (mean ± SD, images *n* = 8, from 4 biologically independent samples). Nuclei were counterstained with Hoechst (blue). Scale bars, 40 μm. Unpaired two-sided Student’s *t-*test (**c**–**e**, **h**), Welch’s two-sided *t-*test (**k**). Source data are provided as Source Data file.

To explore the functional roles of PHB in GSCs, we utilized the CRISPR-Cas9 system to knock out (KO) *PHB* genes in different GSC lines. KO of *PHB* using two independent small guide RNAs (sgRNAs) effectively deleted endogenous PHB expression and significantly decreased the levels of SOX2 and Olig2 in GSCs (Fig. 2a and Supplementary Fig. 3a). These results were validated with PHB knockdown (KD) by expressing doxycycline (DOX)-inducible small hairpin RNAs (DOX-induced shRNAs) in GSCs (Fig. 2b). The downregulation of SOX2 and Olig2 appeared to occur primarily at the post-transcriptional level, as we did not observe a significant decrease in mRNA expression of SOX2 and Olig2 in PHB-deleted GSCs (Supplementary Fig. 3b). Notably, KO or inducible-KD of PHB strongly inhibited GSC viability (Supplementary Fig. 3c, d), decreased GSC self-renewal capacity as demonstrated by sphere formation assays (Fig. 2c, d) and in vitro limiting dilution assays (LDA) (Fig. 2e). However, PHB KD did not remarkably affect the growth of NHA, NPCs, or NSTCs (Fig. 2f and Supplementary Fig. 3e), and this may be due to the limited expression of PHB in these cells. As a control, DOX treatment did not cause any changes in stem cell marker expression or cell growth in control GSCs (Supplementary Fig. 3f).

GSCs possess a strong capacity for self-renewal to seed new tumors^9,39^. To explore the role of PHB in GSC tumor initiation, we established orthotopic GBM xenografts by transplanting control or *PHB* KO GSCs into brains of immunocompromised mice. When the first mouse developed neurological signs, a subset of mice in each group was sacrificed for histological analysis. Mice in the control group developed large tumors, while smaller tumors were found in mice bearing *PHB* KO GSCs (Supplementary Fig. 3g). Consequently, mice implanted with *PHB* KO GSCs exhibited significantly prolonged survival (Fig. 2g). To modulate PHB expression in vivo, GSCs stably expressing luciferase and DOX-induced PHB shRNAs (Luc/DOX-shPHB) were intracranially injected into mice, which were then treated with or without DOX from day 0. Inducible KD of PHB in vivo markedly inhibited GSC tumorigenesis, as indicated by bioluminescence monitoring of tumor response (Fig. 2h), and prolonged animal survival (Fig. 2i). However, overexpression of PHB did not significantly affect SOX2 expression, tumorsphere formation, or tumorigenesis of NSTCs (Supplementary Fig. 3h, i).

To assess whether the impact of PHB depletion on tumor growth is due to the inhibition of GSC self-renewal, we performed an in vivo serial transplantation assay, the gold standard for evaluation of CSCs self-renewal^6,22,40^. Briefly, different numbers of GSCs (5 × 10^4^ or 5 × 10^3^) were implanted into the brains of mice. All mice implanted with control GSCs developed tumors in the primary and secondary xenografts. In the 5 × 10^4^ GSCs groups, the difference in median survival between the first transplantation in control and *PHB* KO groups was 10 days, which was significantly extended to 28 days upon the 2nd transplantation (Fig. 2j, black lines). Notably, in the 5 × 10^3^ GSCs groups, *PHB* KO prolonged the median survival of mice bearing GSCs to 14 days in the 1st transplantation. However, only two tumors were observed in mice with the 2nd transplantation of *PHB* KO GSCs (Fig. 2j, red lines). Meanwhile, we found that both *PHB* KO and PHB inducible-KD resulted in a pronounced reduction of SOX2+ or Olig2+ tumor cells in GBM xenografts (Fig. 2k and Supplementary Fig. 3j, k), suggesting a decrease of GSC pool in PHB-deleted tumors. These results demonstrate that deletion of PHB compromises GSC self-renewal and tumor progression, suggesting that PHB could be a specific therapeutic target for GBM treatment.

### PHB specifically mediates low levels of mitochondrial ROS in GSCs

**Fig. 3:**
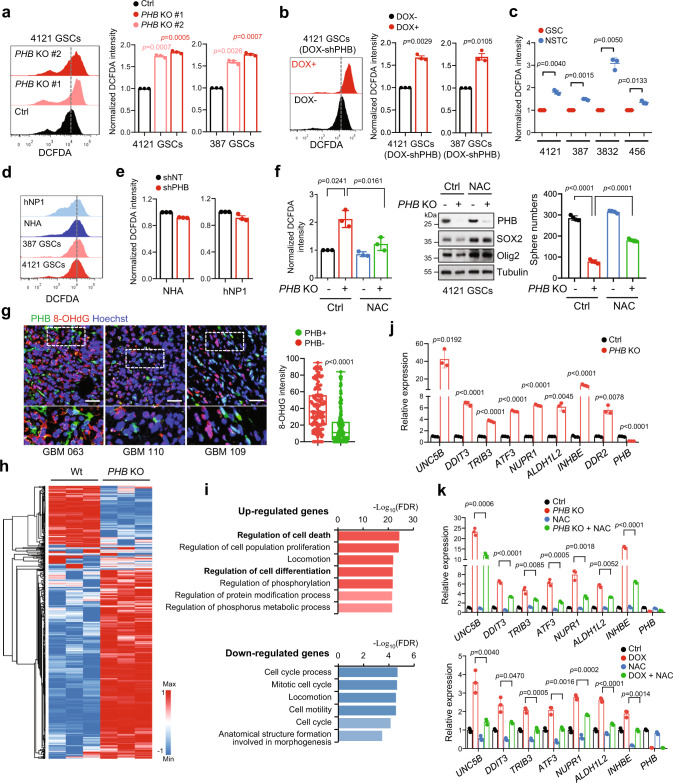
PHB specific mediates low levels of mitochondrial peroxide in GSCs. **a**–**e** The peroxide levels, as indicated by DCFDA fluorescence, were measured by flow cytometry in *PHB* KO GSCs (**a**), PHB inducible-KD GSCs (**b**), GSCs and matched NSTCs (**c**), GSCs, hNP1 and NHA (**d**), or PHB KD NHA and hNP1 (**e**) (mean ± SD, *n* = 3, biologically independent experiments). **f** Ctrl or *PHB* KO 4121 GSCs were treated with vehicle control or NAC (5 mM) for 36 h. Flow cytometry analysis of peroxide by DCFDA staining is shown (left). IB of PHB, SOX2, and Olig2 levels are shown (middle). Quantifications of tumorsphere numbers (2000 cells/well) formed by GSCs are shown (right) (mean ± SD, *n* = 3, biologically independent experiments). **g** Co-IF staining of PHB (green) and 8-OHdG (red) in primary GBM specimens are shown. Quantifications of 8-OHdG staining intensity in PHB− (*n* = 139) and PHB+ (*n* = 127) cells are shown (right). (Boxplots represent the median, 25th, and 75th percentiles). Nuclei were counterstained with Hoechst (blue). Scale bars, 40 μm (up), 20 μm (down). **h** and **i** RNA-seq analysis in control and *PHB* KO 4121 GSCs. In **h**, the heatmap shows relative expression levels of genes downregulated or upregulated in the indicated cells (*p* < 0.05, FC > 2). It includes, respectively, 185 and 740 genes downregulated and upregulated in *PHB* KO compared to control GSCs. Raw data were log_2_ transformed. A relative color scheme used the minimum and maximum values in each row to convert values to colors. In **i**, overrepresented Gene Ontology (GO) terms from RNA-seq analysis of upregulated gene sets (top) and downregulated gene sets (bottom) in *PHB* KO compared to control GSCs. **j** Quantitative real-time PCR (Q-PCR) analysis of mRNA levels of indicated genes in Ctrl and *PHB* KO 4121 GSCs (mean ± SD, *n* = 3, biologically independent experiments). **k** Q-PCR analysis of mRNA levels of indicated genes in *PHB* KO (top) and PHB inducible-KD (bottom) 4121 GSCs treated with vehicle control or NAC (5 mM) (mean ± SD, *n* = 3, biologically independent experiments). Welch’s two-sided *t-*test (**a**–**c**, **e**–**g**, **j**, **k**), Unpaired two-sided Student’s *t-*test (**f**, right). Source data are provided as Source Data file.

To explore the molecular mechanisms by which PHB promotes GSC self-renewal, we first examined the subcellular localization of PHB, as this may be involved in its diverse functions in different tissues^24,25,41^. The ectopically expressed PHB-GFP was co-localized with a mitochondrial protein TOM20 in GSCs (Supplementary Fig. 4a). Co-IF staining of PHB and TOM20 or TIM23 (a mitochondrial protein) showed that endogenous PHB was mainly localized in the mitochondria of GSCs, NSTCs, NPC, and NHA (Supplementary Fig. 4b). Interestingly, we did not observe any change in oxidative phosphorylation (OXPHOS) or ATP generation in mitochondria of PHB-deleted GSCs (Supplementary Fig. 4c, d). However, *PHB* KO significantly increased peroxide production, as assessed with the redox-sensitive probe H_2_DCFDA (Fig. 3a and Supplementary Fig. 4e), and this was further confirmed by PHB inducible-KD in GSCs (Fig. 3b). Redox status plays crucial role in the maintenance and therapeutic resistance of CSCs. Compared to NSTCs, CSCs maintain low levels of ROS and exhibit redox patterns similar to the corresponding normal stem cells^15,17,42^. Consistently, we found that mitochondrial peroxide levels were lower in GSCs than they were in NSTCs (Fig. 3c); however, they were comparable among GSCs, NHA, and NPCs (Fig. 3d). Notably, PHB depletion had no significant effect on the peroxide levels in NHA or NPCs (Fig. 3e). These results reveal that PHB may specifically maintain low levels of mitochondrial ROS in GSCs, suggesting that different mechanisms are involved in ROS regulation between GSCs and NPCs.

To determine whether PHB promotes GSC self-renewal through regulating mitochondrial ROS production, *PHB* KO or PHB inducible-KD GSCs were treated with N-acetyl-l-cysteine (NAC), an ROS-scavenging agent. Treatment with NAC significantly abolished the induction of peroxide by PHB deletion, and this subsequently rescued the reduction of stem cell marker expression and the inhibition of tumorsphere formation due to loss of PHB in GSCs (Fig. 3f and Supplementary Fig. 4f). To further explore the correlation between PHB expression and cell oxidative stress in vivo, we performed Co-IF staining for PHB and 8-OHdG, a marker of DNA oxidative modification by ROS^43^, in human GBM specimens. Tumor cells with high PHB expression displayed significantly lower levels of 8-OHdG immunoreactivity, suggesting the suppression of ROS by PHB (Fig. 3g). Our data also revealed that GSCs contained low levels of ROS in GBM specimens (Supplementary Fig. 4g). The induction of ROS was further confirmed in *PHB* KO and PHB inducible-KD GBM xenografts (Supplementary Fig. 4h, i).

To understand how mitochondrial ROS induced by PHB loss regulates GSC maintenance, we performed RNA-sequencing analysis in control and *PHB* KO GSCs. PHB deletion altered (mostly upregulated) the expression of a large number of genes in GSCs (Fig. 3h and Supplementary Table S2). Gene ontology (GO) analysis of the differentially expressed genes revealed that *PHB* KO resulted in a significant upregulation of genes involved in the regulation of cell death and cell differentiation (Fig. 3i and Supplementary Fig. 4j). Upregulation of genes, such as *ATF3*, *UNC5B*, *DDIT3*, *TRIB3*, *NUPR1* (cell death regulation), *INHBE*, *DDR2* (cell differentiation regulation), *ATF3* and *ALDH1L2* (responding to oxygen-containing compound stimulus) was confirmed by Q-PCR in *PHB* KO and PHB inducible-KD GSCs (Fig. 3j and Supplementary Fig. 4k). Importantly, the induction of these genes was significantly rescued by NAC treatment in GSCs with *PHB* KO or PHB inducible-KD (Fig. 3k), further supporting that PHB deletion induces mitochondrial ROS production, that subsequently increases the gene expression signature involved in regulating cell differentiation and cell death. Collectively, our data suggest that the low mitochondrial ROS status in GSCs is specifically mediated by high levels of PHB, which promotes GSC self-renewal.

### PHB associates with and stabilizes PRDX3 by inhibiting its ubiquitin–proteasome degradation

**Fig. 4:**
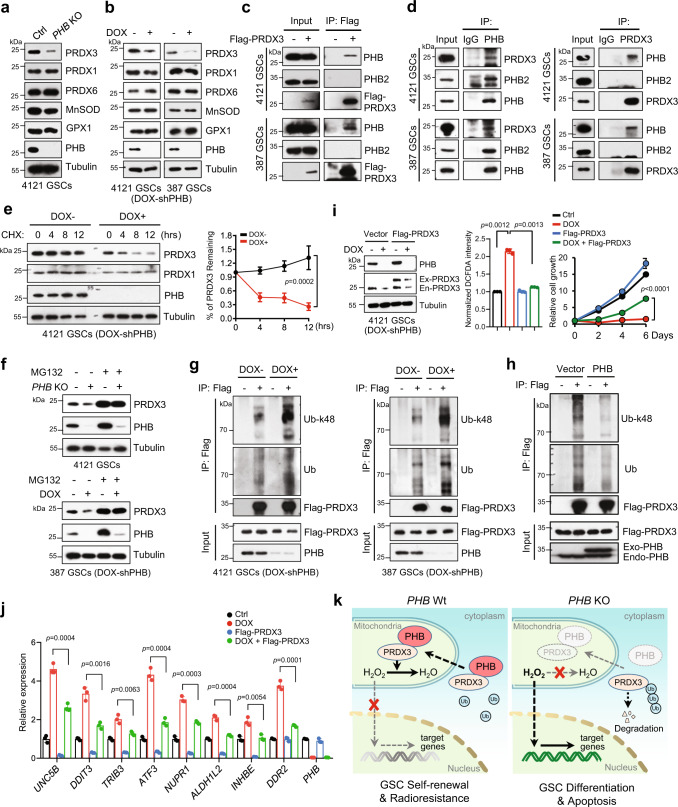
PHB associates with and stabilizes PRDX3 by inhibiting its ubiquitin–proteasome degradation. **a**, **b** IB showing levels of indicated proteins in GSCs with *PHB* KO (**a**) or PHB inducible-KD (DOX, 100 ng/ml) (**b**). **c** Co-immunoprecipitation (Co-IP) with anti-Flag M2 beads in Flag-vector and Flag-PRDX3 expressing GSCs and IB for PHB, PHB2, and PRDX3 are shown. **d** Co-IP with anti-PHB (left) or anti-PRDX3 (right) antibody in GSCs and IB for PHB, PHB2, and PRDX3 are shown. Immunoglobulin G (IgG) was used as a control antibody for IPs. **e** IB showing the CHX (50 μg/ml) chase analysis of PRDX3 protein degradation at indicated time points in GSCs with or without PHB inducible-KD (DOX, 100 ng/ml). Quantifications of relative protein levels of PRDX3 are shown (right) (mean ± SEM, *n* = 5 (0, 4, 8 h) or *n* = 3 (12 h), biologically independent experiments, Two-way ANOVA). **f** IB showing the levels of PRDX3 and PHB in *PHB* KO (top) or PHB inducible-KD GSCs (bottom) treated with vehicle control or MG132 (10 μM) for 12 h. **g** Flag-PRDX3 expressing GSCs with or without PHB inducible-KD (DOX, 100 ng/ml) were treated with MG132 (10 μM) for 12 h. Cell lysates were immunoprecipitated with anti-Flag antibody and IB with anti-ubiquitin-Lys48 (Ub-k48) or anti-ubiquitin (Ub) antibody. **h** IB showing that overexpression of PHB inhibits ubiquitination of PRDX3 in GSCs (4121). **i** Ectopic expression of Flag-PRDX3 rescued the induction of peroxide levels and the inhibition of cell growth by PHB depletion in GSCs. IB showing the levels of PRDX3 and PHB in 4121 GSCs (left). The peroxide levels, as indicated by DCFDA fluorescence, were measured by flow cytometry (middle, mean ± SD, *n* = 3, biologically independent experiments, Welch’s two-sided *t-*test). Cell growth of GSCs was assessed by cell viability assay (right, *n* = 3, biologically independent experiments, two-way ANOVA). **j** Q-PCR analysis of mRNA levels of indicated genes in Ctrl or PHB inducible-KD GSCs (4121) with Flag-vector or Flag-PRDX3 overexpression (mean ± SD, *n* = 3, biologically independent experiments, unpaired two-sided Student’s *t-*test). **k** Proposed model for PHB-PRDX3-mediated regulation of mitochondrial ROS homeostasis. In GSCs, PHB is highly expressed, associated with and stabilizes PRDX3 to maintain mitochondrial ROS homeostasis. Loss of PHB increases PRDX3 ubiquitin–proteasome degradation, elevates ROS levels, and subsequently triggers gene expression to induce GSC differentiation and death. Source data are provided as Source Data file.

We next investigated how PHB modulates mitochondrial peroxide production in GSCs. A study suggests that PHB depletion increases ROS levels via the inhibition of mitochondrial complex I in endothelial cells^44^. However, we did not observe a significant difference in complex I activity between the control and *PHB* KO GSCs (Supplementary Fig. 5a). Mitochondrial ROS levels are tightly controlled by robust scavenger antioxidant enzymes, such as MnSOD (SOD2), glutathione peroxidase (GPX), and PRDXs^13,19,45^. Thus, we examined whether PHB deletion affects the expression of these scavenger enzymes. *PHB* KO remarkably decreased the protein levels of PRDX3, but not those of MnSOD, GPX1, or other PRDX family members such as PRDX1 and PRDX6 in GSCs (Fig. 4a). These results were confirmed by PHB-inducible KD in different GSC lines (Fig. 4b). Moreover, *PHB* KO exerted no impact on the mRNA expression of PRDX3 (Supplementary Fig. 5b), suggesting that PHB may regulate PRDX3 protein stability. However, PHB depletion slightly decreased PRDX3 levels in NSTC, hNP1, and NHA (Supplementary Fig. 5c). We also assessed other antioxidants, including CuZnSOD, GSH, and catalase, and we found that PHB deletion or depletion exerted no significant effect on the levels or activities of these enzymes in GSCs (Supplementary Fig. 5d–f).

PRDX3, a 2-cysteine (Cys) thiol reductase of the PRDX family, is localized in mitochondria. Studies suggest that PRDX3 contributes to nearly 90% of mitochondrial peroxide elimination^19,22^. We observed that PHB was colocalized with PRDX3 in the mitochondria of GSCs (Supplementary Fig. 5g). To assess whether PHB binds to PRDX3, we performed co-immunoprecipitation (Co-IP) with Flag-PRDX3 and found that endogenous PHB interacted with Flag-PRDX3 in GSCs (Fig. 4c). The association between endogenous PRDX3 and PHB was further validated by Co-IP with anti-PHB or anti-PRDX3 antibody (Fig. 4d). PHB was found to interact with its homolog PHB2 (Fig. 4d, left), and this was consistent with a previous report^46^. Interestingly, our results showed that PRDX3 was associated with PHB, but not with PHB2 (Fig. 4c, d), suggesting that different PHB complexes might exist in the mitochondria. We further mapped the interaction regions of these two proteins, and we found that the N-terminal of PRDX3 and the PHB/Coiled-coil domain of PHB were required for their association (Supplementary Fig. 5h, i).

To determine whether PHB regulates PRDX3 protein stability, we performed cycloheximide (CHX) chase assay to examine the proteolytic turnover of PRDX3 in control and PHB-depleted GSCs. We found that PRDX3 protein remained quite stable, as CHX treatment for 12 h did not noticeably impact its stability. However, the half-life of PRDX3 protein, but not other PRDX family members such as PRDX1, was significantly decreased in PHB-depleted GSCs (Fig. 4e and Supplementary Fig. 5j). To assess the potential mechanisms associated with PRDX3 degradation, we treated GSCs with MG132, an inhibitor of the proteasome, or chloroquine, an inhibitor of lysosomal acidification. The results showed that treatment with MG132, but not chloroquine, increased the protein levels of PRDX3 and PHB in a time-dependent manner (Supplementary Fig. 5k). Notably, MG132 treatment largely rescued the decrease in PRDX3 protein in *PHB* KO or PHB inducible-KD GSCs (Fig. 4f), suggesting that the ubiquitin–proteasome pathway may be required for PRDX3 reduction induced by PHB deletion. Indeed, the Lys 48-linked poly-ubiquitination and total poly-ubiquitination of PRDX3 were dramatically increased in GSCs with PHB inducible-KD (Fig. 4g). Overexpressing PHB resulted in a decrease in the Lys 48-linked poly-ubiquitination and total poly-ubiquitination of PRDX3 (Fig. 4h). These data suggest that PHB promotes the stability of PRDX3 protein through the ubiquitin-proteasome pathway.

We next tested whether the functions of PHB are mediated by PRDX3 in GSCs. KD of PRDX3 decreased SOX2 and Olig2 expression, strongly induced peroxide levels, and inhibited cell growth in GSCs (Supplementary Fig. 5l–n). Importantly, overexpression of PRDX3 largely rescued the induction of peroxide and the inhibition of cell growth by PHB depletion in GSCs (Fig. 4i and Supplementary Fig. 5o). Additionally, the induction of indicated gene expression caused by PHB depletion was also significantly rescued by PRDX3 overexpression (Fig. 4j), supporting the crucial role of PRDX3 in PHB signaling. We also confirmed that PHB deletion decreased PRDX3 levels in GSC-derived xenografts (Supplementary Fig. 5p). Collectively, these results suggest that PHB maintains low levels of mitochondrial ROS through binding to PRDX3 and suppressing its ubiquitin–proteasome degradation in GSCs (Fig. 4k).

### PHB promotes GSC radio-resistance

**Fig. 5:**
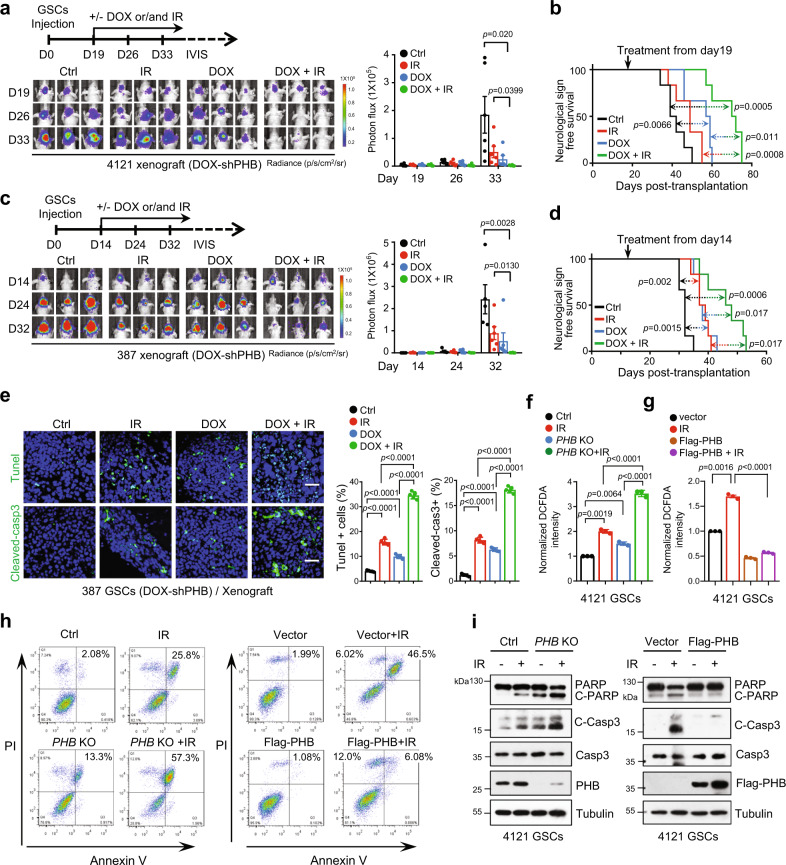
PHB promotes GSC radio-resistance. **a**–**d** Nude mice (nu/nu) were intracranially implanted with GSCs (**a**, **b**, 4121 GSCs; **c**, **d** 387 GSCs) transduced with Luciferase/Tet-on-inducible-shPHB. Mice were randomly grouped (*n* = 6 for each group) and treated with control, IR (3 Gy, once a week, 4 times), DOX (2 mg/ml in drinking water), or the combined treatment from day 19 (**a**, **b**) or day 14 (**c**, **d**) after implantation, as shown by schematic representation (**a**, **c** top). GBM xenografts were tracked by bioluminescence and the representative images are shown (**a**, **c** bottom). Bioluminescent quantifications of tumor growth are shown (**a**, **c** right, mean ± SEM, unpaired two-sided Student’s *t-*test). Kaplan–Meier survival plots of mice are shown (**b**, **d**; Log-rank Mantel–Cox test). **e** IF staining of Tunel (top) or cleaved-caspase3 (bottom) in GBM xenografts from (**c**) are shown (left). Quantifications of Tunel+ or cleaved-caspase3+ cells are shown (right) (mean ± SD, *n* = 5, biologically independent samples, Unpaired two-sided Student’s *t-*test). Scale bars, 40 μm. **f**, **g** The peroxide levels, as indicated by DCFDA fluorescence, were measured by flow cytometry in 4121 GSCs with indicated treatments. IR, 3 Gy for 48 h (**f**) or 72 h (**g**) (mean ± SD, *n* = 3, biologically independent experiments, Welch’s two-sided *t-*test). **h** Cell apoptosis was measured by flow cytometry in 4121 GSCs with indicated treatments. IR, 3 Gy for 48 h (left) or 72 h (right). **i** IB showing levels of cleaved-PARP, cleaved-caspaspe3, caspase3, and PHB in GSCs with indicated treatments. IR, 3 Gy for 48 h (left) or 72 h (right). Source data are provided as Source Data file.

It is well recognized that ROS is a critical mediator of IR-induced cell killing. IR directly causes DNA double-strand breaks or indirectly induces DNA damage by increasing ROS production^16,47^. To investigate whether the ROS induced by PHB loss sensitizes GBM to radiotherapy, we established the orthotopic GBM xenografts using GSCs expressing Luc/DOX-shPHB. When tumors reached a similar size (day 19 after 4121 GSCs transplantation, or day 14 after 387 GSCs transplantation) (Fig. 5a, c, up), mice were grouped randomly and treated with control, IR (3 Gy, once every week, four times in total), DOX (daily in water) or IR plus DOX (Fig. 5a, c, up). The results showed that abrogating PHB expression potently inhibited tumor growth in the established GBM models (Fig. 5a, c) and extended survival (Fig. 5b, d). IR monotherapy showed limited (Fig. 5a, b) or effective (Fig. 5c, d) suppressive effects on the growth of the established tumors and slightly extended survival. Importantly, losing PHB expression significantly sensitized tumors to IR treatment (Fig. 5a, c) and conferred the longest survival extension among all experimental groups (Fig. 5b, d), highlighting targeting PHB as a therapeutic index for GBM combination therapy. In contrast, we did not observe a significant impact of DOX treatment on the sensitivity of GSCs to IR in vitro or in vivo (Supplementary Fig. 6a, b).

TUNEL or cleaved-caspase3 staining showed that, whereas suppression of PHB expression or IR treatment alone increased tumor cell apoptosis in vivo, the combination treatment resulted in much more cell death in tumors (Fig. 5e), confirming that PHB depletion sensitizes GBM to IR treatment. In addition, IR treatment-induced peroxide production in GSCs in vitro, which was significantly increased by combination with PHB deletion (Fig. 5f and Supplementary Fig. 6c). However, overexpression of Flag-PHB decreased the accumulation of peroxide-induced by IR in GSCs (Fig. 5g and Supplementary Fig. 6d). Furthermore, abrogation of PHB remarkably increased GSC apoptosis induced by IR in vitro, as evaluated by flow cytometry analyses of Annexin V and propidium iodide (PI) (Fig. 5h and Supplementary Fig. 6e, left), or by IB analysis with cleaved-caspase3 and cleaved-PARP (Fig. 5i and Supplementary Fig. 6f, left). Overexpression of Flag-PHB strongly decreased GSC apoptosis induced by IR (Fig. 5h, i, and Supplementary Fig. 6e, f, right). Notably, the synergistic effect of *PHB* KO and IR on peroxide induction and cell killing was partially rescued by NAC treatment in vitro (Supplementary Fig. 6g, h).

We observed that IR treatment resulted in an increase of DNA damage in GSCs, as assessed by phospho-histone 2A.X (γH2AX), a marker of DNA double-strand breaks (Supplementary Fig. 6i, left). *PHB* KO elevated the γH2AX levels even in the absence of IR treatment, suggesting the induction of DNA damage by PHB deletion. Importantly, PHB deletion strongly increased, and Flag-PHB overexpression decreased the DNA damage induced by IR (Supplementary Fig. 6i). Moreover, depletion of PHB compromised the repair of DNA damage induced by IR in GSCs (Supplementary Fig. 6j), and this may contribute to the sensitization of GSCs to radiotherapy. In addition to radiotherapy, chemotherapy using the DNA alkylating agent temozolomide (TMZ) is another standard treatment for GBM^4^. Our data showed that PHB depletion also increased the sensitivity of GSCs to TMZ treatment; however, the synergistic effect was weaker than that of the combined treatment of PHB depletion and IR (Supplementary Fig. 6k). Taken together, these data suggest that high levels of PHB protect GSCs from IR-induced cell death through reducing ROS accumulation and that abrogating PHB synergizes with radiation to improve GBM treatment.

### Pharmacological targeting of PHB inhibits GSC self-renewal and sensitizes GBM to radiotherapy

**Fig. 6:**
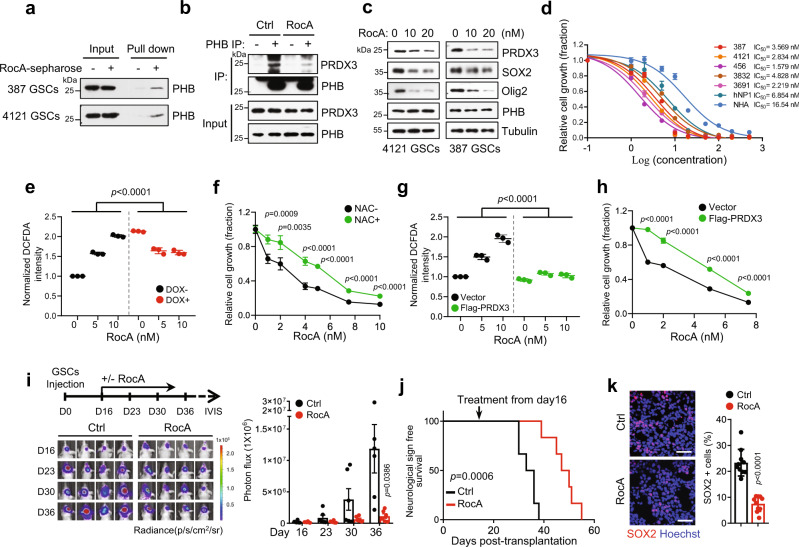
Pharmacological targeting PHB inhibits GSC growth and tumorigenesis. **a** The association of RocA and PHB was analyzed by RocA-conjugated sepharose pull-down in GSCs. **b** Co-IP of PHB in 4121 GSCs treated with RocA (10 nM) and MG132 (10 μM) for 12 h and IB for PHB and PRDX3 are shown. **c** IB showing the levels of indicated proteins in GSCs treated with increasing dose of RocA for 12 h. **d** Dose–response curves of RocA treatment in multiple GSC lines, hNP1 and NHA. Cells were treated with increasing dose of RocA for 48 h. IC_50_ values of RocA were measured using nonlinear regression analysis of dose–response curves (mean ± SD, *n* = 3, biologically independent experiments). **e**, **g** The peroxide levels, as indicated by DCFDA fluorescence, were measured by flow cytometry in Ctrl or PHB inducible-KD 4121 GSCs (DOX, 100 ng/ml) (**e**), or in Flag-vector or Flag-PRDX3 expressed 4121 GSCs (**g**) treated with increasing dose of RocA for 18 h (mean ± SD, *n* = 3, biologically independent experiments, two-way ANOVA). **f**, **h** 4121 GSCs cultured with or without 5 mM NAC (**f**), or expressed with Flag-vector or Flag-PRDX3 (**h**), were treated with indicated doses of RocA for 3 days. Cell growth was assessed by cell viability assay. Data were normalized to the untreated cells of each group (mean ± SD, *n* = 4 (**f**) or *n* = 3 (**h**), biologically independent experiments, unpaired two-sided Student’s *t-*test, no adjustment). **i**–**k** Nude mice (nu/nu) intracranially implanted with 4121 GSCs (Luciferase) were randomly grouped (*n* = 6) at day 16 and treated with or without RocA (2.5 mg/kg, every 3 days, 8 times in total), as shown by schematic representation (**i**, top). GBM xenografts were tracked by bioluminescence and the representative images are shown (**i**, left). Bioluminescent quantification of tumor growth is shown (**i**, right; mean ± SEM, Welch’s two-sided *t-*test). Kaplan–Meier survival plot of mice is shown (**j**, Log-rank Mantel–Cox test). IF staining of SOX2 in GBM xenografts with indicated treatments and the representative images are shown (**k**, left). Quantifications of SOX2+ cells are shown (**k**, right) (mean ± SD, images *n* = 10, from five biologically independent samples, Unpaired two-sided Student’s *t*-test, no adjustment). Nuclei were counterstained with Hoechst (blue). Scale bars, 40 μm. Source data are provided as Source Data file.

Previous studies have shown that rocaglamide A (RocA), a compound isolated from the genus *Aglaia*, targets PHB directly by blocking the interaction between PHB and its binding proteins^48,49^. Consistent with this, we found that RocA bound to endogenous PHB in GSCs (Fig. 6a) and efficiently blocked the association between PHB and PRDX3 in GSCs (Fig. 6b and Supplementary Fig. 7a). Consequently, the expression of PRDX3 and stem cell markers was decreased upon RocA treatment in a time-dependent and dose-dependent manner (Fig. 6c and Supplementary Fig. 7b). We then tested the effect of RocA on GSC maintenance, and we found that RocA treatment caused a dose-dependent inhibition of cell growth in multiple GSC lines (Supplementary Fig. 7c). The half-maximal inhibitory concentration (IC_50_) analysis showed that a very low concentration of RocA (<5 nM) achieved significant suppression of GSC growth (Fig. 6d). However, RocA treatment showed relatively low toxic effects on NHA, NPCs, NSTCs, and the established glioma cell lines at the effective dose for GSCs (Supplementary Fig. 7d–f).

To determine whether the impact of RocA on GSCs is mediated through inhibition of PHB-PRDX3 signaling, we treated control and PHB-inducible KD GSCs with RocA. RocA treatment strongly induced peroxide production in a dose-dependent manner in GSCs (Fig. 6e, g, left). However, RocA did not increase peroxide levels in PHB-depleted GSCs as much as it did in control GSCs (Fig. 6e), suggesting an important role of PHB in ROS induction by RocA. Notably, scavenging peroxide by NAC significantly rescued the inhibition of GSC growth by RocA (Fig. 6f). As RocA treatment resulted in the dissociation of PRDX3 from PHB and a subsequent decrease in PRDX3 protein levels (Fig. 6b, c and Supplementary Fig. 7a, b), we ectopically expressed Flag-PRDX3 in GSCs, and we found that overexpression of PRDX3 potently compromised the induction of peroxide by RocA (Fig. 6g), and largely rescued the inhibitory effect of RocA on GSC growth (Fig. 6h).

Studies suggest that some potential targets, such as NF-κB, c-Raf, and eIF4A may be involved in the anti-cancer activity of RocA^48,50,51^. We further assessed the effects of RocA on these signaling pathways in GSCs. Our results showed that RocA treatment at the same concentration as that for growth inhibition in GSCs caused no obvious impact on the activation of NF-κB and c-Raf-ERK signaling pathways (Supplementary Fig. 7g, h). Many transcriptional factors and oncogenes, including c-Myc, EZH2, Notch1, and Bcl2, require eIF4A for translation^52^ (Supplementary Fig. 7i, left). However, RocA treatment did not decrease these proteins in GSCs (Supplementary Fig. 7i, right). Together, our results suggest that RocA induces mitochondrial ROS levels and suppresses GSC growth mainly through inhibiting the PHB–PRDX3 pathway. We further investigated the effect of fluorizoline, a synthetic molecule that binds to and targets PHB^53^, on GSCs. Consistent with the effect of RocA on GSCs, fluorizoline treatment decreased the expression of PRDX3 and stem cell markers in GSCs (Supplementary Fig. 7j), and inhibited GSC growth in a dose-dependent manner (Supplementary Fig. 7k, left). However, fluorizoline also showed a remarkable suppression on hNP1 and NHA (Supplementary Fig. 7k, right), indicating that the undefined targets of fluorizoline may exist. Moreover, treatment with fluorizoline significantly inhibited the established GBM tumor growth and extended survival (Supplementary Fig. 7l).

To justify the RocA use in GBM treatment in vivo, we intraperitoneally injected RocA into normal mice or mice bearing orthotopic GBM xenografts and assessed its bioavailability in mouse brains. The mass spectrometry analysis showed that the concentration of RocA was significantly increased in mouse brains and was higher in brain tumor tissues than that was in normal brains (Supplementary Fig. 8a, b), suggesting that RocA could penetrate mouse brain tumors to exert tumoricidal effects. Importantly, RocA administration remarkably suppressed the growth of the established tumors (Fig. 6i) and conferred a significant survival benefit (Fig. 6j). Moreover, the inhibitory effects of RocA on GSC-derived xenografts were rescued by overexpressing PRDX3 in GSCs (Supplementary Fig. 8c). Consistent with the results from PHB deletion in GSC-derived xenografts, RocA treatment resulted in a dramatic increase in ROS levels, a reduction in SOX2+ tumor cells, and an increase in cell death in tumors (Fig. 6k and Supplementary Fig. 8d). Notably, RocA treatment did not show detectable toxic effects in vivo, as it did not cause any weight loss in mice (Supplementary Fig. 8e), morphological changes in liver and lung tissues assessed by H&E staining (Supplementary Fig. 8f), or alterations in the survival of NPCs residing in the subventricular zone (SVZ) of mouse brains (Supplementary Fig. 8g). These data suggest that RocA treatment selectively targets GSCs and inhibits the growth of orthotopic GBM xenografts but has no severe toxicity on normal tissues.

**Fig. 7:**
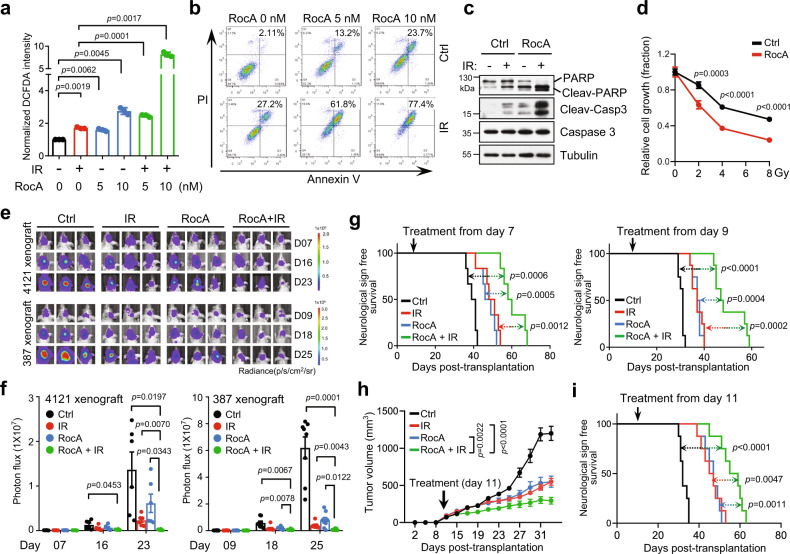
Pharmacological targeting PHB increases sensitiveness of GSCs to IR. **a** The peroxide levels, as indicated by DCFDA fluorescence, were measured by flow cytometry in 4121 GSCs with indicated treatment for 48 h. IR, 3 Gy (mean ± SD, *n* = 3, biologically independent experiments, Welch’s two-sided *t-*test). **b** Cell apoptosis was measured by flow cytometry in 4121 GSCs with indicated treatments for 48 h. IR, 3 Gy. **c** IB showing levels of cleaved-PARP, cleaved-caspaspe3, and caspase3 in 4121 GSCs with indicated treatments for 48 h. IR, 3 Gy. **d** Cell growth of 4121 GSCs treated with indicated doses of IR for 48 h in the absence or presence of RocA (5 nM). Data were normalized to the untreated cells of each group (mean ± SD, *n* = 4, biologically independent experiments, unpaired two-sided Student’s *t-*test). **e–g** Nude mice (nu/nu) intracranially implanted with GSCs (Luciferase) were randomly grouped (4121 GSCs, *n* = 6; 387 GSCs, *n* = 8, for each group) and treated with control, IR (3 Gy, once a week, three times), RocA (2.5 mg/kg, every 3 days, six times), or the combined treatment from day 7 (4121 GSC xenografts) or day 9 (387 GSC xenografts). GBM xenografts were tracked by bioluminescence and the representative images are shown (**e**). Bioluminescent quantifications of tumor growth are shown (**f**) (mean ± SEM, Welch’s two-sided *t-*test). Kaplan–Meier survival plots of mice are shown (**g**, 4121 GSCs, left; 387 GSCs, right) (Log-rank Mantel–Cox test). **h**, **i** NOD/SCID mice were subcutaneously implanted with GBM PDX tumors. Mice were randomly grouped (*n* = 8 for each group) at day 11 and treated with control, IR (3 Gy, once a week, 4 times), RocA (2.5 mg/kg, every 3 days, eight times in total) or the combined treatment. Tumor volume was measured (**h**) (mean ± SEM, Two-way ANOVA). Kaplan–Meier survival plot of mice is shown (**i**). (Log-rank Mantel–Cox test). Source data are provided as Source Data file.

We then assessed whether targeting PHB by RocA synergizes with IR in GBM treatment. Our results showed that combined RocA and IR treatment dramatically induced peroxide levels (Fig. 7a and Supplementary Fig. 9a) and increased cell apoptosis in GSCs (Fig. 7b, c and Supplementary Fig. 9b,c). Furthermore, RocA treatment sensitized GSCs to IR treatment in vitro (Fig. 7d). Importantly, in two established orthotopic GBM xenografts, the combination of RocA and IR treatment yielded the greatest efficacy in suppressing tumor growth and extending overall survival relative to RocA or IR treatment alone (Fig. 7e–g). The therapeutic efficacy of the combination treatment was further validated in GBM patient-derived xenografts (PDXs) (Fig. 7h, i). Meanwhile, TUNEL staining showed that the combination treatment resulted in a remarkable increase in cell death in tumors, whereas RocA or IR alone increased tumor cell apoptosis, validating the synergistic effect of RocA and IR treatment on inhibiting GBM growth (Supplementary Fig. 9d). To further evaluate the potential therapeutic value of RocA in GBM treatment, we compared the tumor inhibitory effects of RocA and TMZ. Our data indicated that RocA was superior to TMZ treatment in inhibiting GSC growth in vitro (Supplementary Figs. 7c, 9e). Moreover, the combined RocA and TMZ treatment did not show a dramatic increase in GSC apoptosis in vitro (Supplementary Fig. 9f), or a strong synergistic therapeutic effect on GBM growth in vivo (Supplementary Fig. 9g). Collectively, these results demonstrate that targeting GSCs by RocA effectively suppresses GSC-derived tumor growth and significantly improves the efficacy of radiotherapy for GBM.

## Discussion

ROS play crucial roles in stem cell maintenance^42,54^. Our results revealed that GSCs maintain low mitochondrial ROS content that is similar to NPCs, when compared to levels in NSTCs. This raises an important question regarding how to eliminate GSCs by targeting the ROS-mediated mechanisms without affecting normal stem cells. In this study, we revealed that PHB is a novel regulator that specifically controls ROS levels in GSCs. PHB is highly expressed in GSCs relative to NPCs, NHA, and NSTCs. Although disruption of PHB strongly increases ROS accumulation and compromises GSC self-renewal both in vitro and in vivo, abrogation of PHB has a weak inhibitory effect on NPCs or NHA growth, highlighting the unique role of PHB in ROS regulation in GSCs.

PHB is an evolutionarily conserved protein that has been implicated in diverse cellular processes, including cell proliferation, replicative senescence, and mitochondrial biogenesis^24,25^. However, how PHB act at the molecular level is not yet fully understood. Studies indicate that PHB may be involved in the regulation of complex I of mitochondrial electron transport chain and in mitochondrial respiration^25,44,55^. However, our data indicated that the respiration and activity of complex I in mitochondria are not significantly impaired by PHB deletion in GSCs, which is consistent with other previous findings^56^, suggesting that the roles of PHB may be cell-type dependent. Our results demonstrated that PHB associates with and stabilizes PRDX3, a peroxiredoxin protein that scavenges peroxides and protects cells from oxidative stress^22^. The function of PHB is likely mediated by PRDX3, as overexpressing PRDX3 or scavenging ROS by NAC rescues the induction of ROS and the inhibition of self-renewal caused by PHB deletion in GSCs. We further demonstrated that PHB protects PRDX3 from ubiquitin–proteasome-dependent degradation; however, the detailed molecular mechanisms require further elucidation.

GBM is the most common primary brain malignancy in adults with no effective treatment. Standard non-surgical treatment, including radio-chemotherapy, produces only modest benefits partially due to the resistance of GSCs^4,7,39^. Understanding the underlying mechanisms of resistance may provide new strategies to improve GBM treatment. We demonstrated that low levels of ROS mediated by PHB protect GSCs from IR-induced cell killing and promote GBM therapeutic resistance. Targeting PHB by genetic deletion or pharmacological inhibition effectively suppresses tumor growth, overcomes the resistance of GSCs to radiotherapy, and thus extends animal survival in preclinical GBM models. Meanwhile, the percentage of PHB+ cells is increased in high-grade gliomas and positively correlated with the recurrence and poor prognosis of glioma patients, reinforcing that PHB may be an attractive target for GBM therapy.

Rocaglamides are natural products that have been observed to possess antitumor activities in various tumor cell lines^57^. Although studies suggest that the primary effect of RocA may be inhibition of protein synthesis^50^, its direct molecular targets remain largely unknown. Recently, a study has shown that RocA directly binds to PHB and blocks the membrane associations between PHB and c-Raf^48^. In our study, we found that RocA binds to PHB and decreases the interaction between PHB and PRDX3. Notably, a very low dose of RocA (<10 nM) recapitulates the findings observed with PHB deletion, including the induction of mitochondrial ROS and the inhibition of cell growth in GSCs with limited toxicity in NHA or NPCs. Meanwhile, the overexpression of PRDX3 largely attenuates the induction of ROS by RocA and blocks its inhibitory effects in GSCs. These findings suggest that the effects of RocA are likely mediated through the inhibition of PHB-PRDX3 signaling in GSCs. Our data revealed that low doses of RocA result in no obvious impact on the c-Raf/ERK, NF-κB, or eIF4A signaling pathways^48,50,51^, suggesting that the action mode of RocA may be cell-type dependent and associated with the subcellular localization of PHB. Most importantly, RocA treatment significantly inhibits GBM tumor growth and provides survival benefits without any detectable signs of sickness or morbidity in tumor-bearing mice. Combination treatment with RocA and radiotherapy represents a strong synergistic anti-tumor effect in established orthotopic GBM xenografts and GBM PDXs, supporting the potential of RocA for clinical application in GBM treatment both as a monotherapy and as an adjuvant therapy with radiation.

## Methods

### Cells, tissues, and cell culture

GSCs were isolated from GBM surgical specimens or xenografts and were functionally characterized^32–35^. Briefly, cells were isolated from primary GBM specimens or patient-derived GBM xenografts according to the Papain Dissociation System (Worthington Biochemical) manufacturer’s instructions and recovered in the stem cell medium (Neurobasal-A medium with B27 supplement, 10 ng/ml EGF, 10 ng/ml βFGF, 1% penicillin/streptomycin, 1 mM sodium pyruvate, and 2 mM l-glutamine) overnight in a humidified incubator with 5% CO_2_. GSCs were then sorted by magnetic cell sorting using the surface marker CD133 (Miltenyi Biotec.) and cultured in stem cell medium as described above and assayed for expression of stem cell markers including SOX2, Olig2, Nestin, and absence of GFAP expression. A series of functional assays were then applied to validate the cancer stem cell phenotypes of the isolated GSCs, including the sphere-forming ability (in vitro limiting dilution assay), multipotent differentiation (10% fetal bovine serum induction of multi-lineage differentiation in vitro), and tumor-initiation in immunocompromised mice in our previous studies^32,33^. Specifically, 4121 GSCs were derived from a recurrent GBM patient (53-year old, male); 387 GSCs were derived from a primary GBM patient (76-year old, female); 3691 GSCs were derived from a primary GBM patient (59-year old, female); 456 GSCs were derived from a primary GBM patient (8-year old, female); 3832 GSCs were derived from a primary GBM patient (75-year old, female).

NSTCs (CD133−) were isolated from patient-derived GBM xenografts and cultured in Dulbecco’s modified Eagle’s medium (DMEM) supplemented with 10% FBS to maintain differentiation status. Low (<5) passage cells were used for experiments to prevent cellular drift. For cell differentiation assay, GSCs were induced for differentiation by the withdrawal of growth factors and by the addition of 10% FBS in DMEM. On the indicated day, cells were harvested for IB analysis.

Three human NPC lines (hNP1, 15167, 17231, derived from fetal brains, Lonza) were cultured and maintained in the stem cell medium described above according to the vendor’s instruction. NHA (Beina Chuanglian Biotechnology Institute) was maintained in glucose-free DMEM supplemented with 5 mM glucose and 10% FBS.

GBM surgical specimens were collected with the approval of the PLA General Hospital. Informed consent was obtained for all subjects. Specifically, G#7 was from a GBM patient (56-year old, male); G#19 was from a GBM patient; G#27 from a GBM patient (56-year old, male); G#28 from a GBM patient (73-year old, female); G#29 from an Astrocytoma patient (Grade III, 53-year old, male); G#32 from a GBM patient. The glioma tissue microarray was purchased from Shanghai Outdo Biotechnology Company, Ltd (HBraG171Su01). Histologic diagnosis of the tissue microarray cores was reviewed by at least two individuals, one of whom is a pathologist. 11 specimens were removed because of tissue incompletion or mismatching with the pathological diagnosis. The tissue microarray included tumors from 94 patients with grade I–II astrocytoma, 35 females, 59 males, ages 3–80 years old, median 41 years; 46 patients with grade III astrocytoma, 19 females, 27 males, ages 17–68 years old, median 43 years; 20 patients with GBM, 3 females, 17 males, ages 9–78 years old, median 46 years. The patients’ information of the tissue microarray is listed in Supplementary Table 1.

### Orthotopic mouse xenografts

All animal experiments were performed in accordance with the NIH guide for the care and use of laboratory animals and with the approval of the Institutional Animal Care and Use Committee of National Center of Biomedical Analysis. Mice used in our studies were 4-week-old female NU/NU nude mice purchased from Beijing Vital River Laboratory Animal Technology. Mice had not undergone prior treatment or procedures. Animal care was monitored daily by certified veterinary staff and laboratory personnel. Five mice were housed per cage, with a 14 h light/10 h dark cycle, and were provided with food and water. Animals were monitored until neurological signs were observed, at which point they were sacrificed by inhalation of carbon dioxide followed by cervical dislocation. All surgical procedures were performed under anesthesia by intraperitoneal injection of a ketamine and xylazine cocktail. Tissues were removed following euthanasia and were fixed in 4% paraformaldehyde (PFA, Sigma-Aldrich) at 4 °C for 24 h, stored in 30% sucrose solution at 4 °C for 48 h, embedded in OCT at −80 °C overnight, and cryosection thickness of 7 microns. Methods describing the establishment of mouse orthotopic xenograft are described below.

### Immunofluorescence staining, immunohistochemistry, and IB

For the Immunofluorescent staining experiments, the cultured cells, tissue cryosections, or human surgical specimens were fixed in 4% PFA for 15 min, and were then permeabilized in PBS containing 0.3% Triton X-100 (Bio-Rad Laboratories) for 15 min. Samples were blocked with 5% albumin from bovine serum with 0.3% Triton X-100 in PBS for 60 min at room temperature and then incubated with primary antibodies overnight at 4 °C followed by the appropriate secondary fluorescently labeled antibodies for one hour at room temperature. Nuclei were counterstained with Hoechst (Invitrogen). Images were acquired using a laser confocal microscope (Zeiss, LSM880) with Zen 2.1 SP2 software, and were processed using Image J software 1.8.0.

For the immunohistochemical staining experiments, a tissue microarray of deidentified formalin-fixed, paraffin-embedded glioma specimens was immunostained with PHB antibody. Secondary antibody was labeled with polymer-HRP (horseradish peroxidase) anti-rabbit as appropriate. Staining was visualized using 3,30-diaminobenzidine (DAB) chromogen (Zhongshan Golden Bridge). Presence or absence of PHB staining was scored by at least two individuals, one of whom is a pathologist, and consensus scores are reported. Briefly, the staining intensity of PHB has assessed both the intensity of the staining and the percentage of positively stained cells. For the intensity, a score of 0–3 (corresponding to negative, weak, moderate, or strong staining) was recorded and the percentage of positively stained cells at each intensity was estimated.

Immunoblotting was carried out following standard methods^32^. Briefly, cells were lysed in lysis buffer (Tris–HCl 20 mM PH 7.4, 0.5% NP-40, 250 mM NaCl, 3 mM EGTA, 3 mM EDTA) supplemented with protease inhibitors (Roche) and incubated on ice for 30 min. The Bradford assay (Bio-Rad Laboratories) was utilized for the determination of protein concentration. Equal amounts of protein were mixed with reducing Laemmli loading buffer, boiled for 10 min, and resolved by SDS–PAGE, then transferred onto PVDF membranes (Millipore, Billerica, MA). Blots were incubated with primary antibodies overnight at 4 °C followed by HRP-conjugated species-specific antibodies (Jackson Immuno Research, 1:5000) at room temperature for one hour.

The following antibodies were used: PHB (Abcam for IB 1:5000; for IF, 1:200; for IHC 1:100), SOX2 (Millipore for IB, 1:1000; Santa Cruz for IF, 1:200), Olig2 (Santa Cruz for IB, 1:1000; R&D for IF, 1:200), GFAP (Dako for IB:1000), PRDX3 (Novus for IB, 1:5000; for IF, 1:200), 8-OHdG (Abcam for IF, 1:400), TOM20 (Santa Cruz for IF, 1:400), TIM23 (Santa Cruz for IF,1:200), Tubulin (Sigma for IB, 1:5000), Caspase3 (Proteintech for IB, 1:1000), Cleaved-Caspase3 (Cell Signaling for IB, 1:1000; for IF, 1:100), PARP (Cell Signaling for IB, 1:1000), Flag (Sigma for IB, 1:2000), PRDX1 (Proteintech for IB, 1:1000), PRDX6 (Proteintech for IB, 1:1000), GPX1 (Abcam for IB, 1:1000), Mn-SOD (Proteintech for IB, 1:1000), PHB2 (Cell Signaling for IB,1:1000), β-Actin (Santa Cruz for IB, 1:1000), Ubiquitin (MBL for IB, 1:1000), Ubiquitin, Lys48-specific (Millipore for IB, 1:1000), p65 (Santa for IB, 1:500), phospho-p65 (Cell Signaling for IB, 1:1000), c-Raf (Cell Signaling for IB, 1:1000), Phospho-c-Raf (Cell Signaling for IB,1:1000), ERK (Cell Signaling for IB, 1:500), Phospho-p44/42 MAPK (Erk1/2) (Cell Signaling for IB, 1:500), eIF4A1 (Cell Signaling for IB, 1:1000), Notch1 (Cell Signaling for IB, 1:1000), c-Myc (Cell Signaling for IB, 1:1000), Bcl-2 (Santa Cruz for IB, 1:500), Ezh2 (Cell Signaling for IB, 1:1000), Cu-ZnSOD (Proteintech for IB, 1:1000), γH2AX (Millipore for IB, 1:1000). Secondary antibody labeled with polymer-HRP anti-rabbit (Jackson for IB, 111-035-003, Lot:147832, 1:5000), secondary antibody labeled with polymer-HRP anti-mouse (Jackson for IB,115-035-003, Lot:148148, 1:5000). Alexa Fluor 488 donkey anti-rabbit IgG (Thermo for IF, A21206, Lot:1927937, 1:400), Alexa Fluor 488 donkey anti-mouse IgG (Thermo for IF, A21202, Lot:1915874, 1:400), Alexa Fluor 555 donkey anti-mouse IgG (Thermo for IF, A31570, Lot:1905844, 1:400), and Alexa Fluor 555 donkey anti-goat IgG (Thermo for IF, A21432, Lot:1932497, 1:400).

### DNA constructs and lentiviral transfection

Human Flag-PHB and Flag-PRDX3 were generated by PCR and were cloned into the pCDH-MCS-T2A-Puro-MSCV lentiviral vectors (System Biosciences). The truncated mutant forms of PHB (1–90aa, 1–173aa, 41–272aa, 174–272aa) were cloned into the pCDH-MCS-T2A-Puro-MSCV vectors. The truncated mutant forms of PRDX3 (1–221aa, 63–221aa, 63–256aa) were cloned into the pGEX-4T-1 vector (Adgene, #27-4580-01). Clones expressing two non-overlapping sgRNAs directed against human *PHB* or a non-targeting control sgRNA that has no targets in the human genome were cloned into the LentiGuide-puro vectors (Addgene); Clones expressing PHB DOX-induced shRNAs were cloned into the Tet-pLKO-puro vectors (Addgene); Clones expressing PHB, PRDX3 or EIF4A shRNAs were cloned into the pLKO.1 TRC vectors (Addgene). The sgRNAs and shRNAs used in this study are listed in Supplementary Table 2.

HEK293 cells were used to generate lentiviral particles through co-transfection of the packaging vectors pSPAX2 and pVSVG (Addgene) using a standard calcium phosphate transfection method. For rescue experiments, GSCs stably expressing Flag-PRDX3 were transduced with PHB shRNAs lentiviral constructs. After recovering for 48 h, cells were selected by puromycin (2 μg/ml).

### CRISPR–Cas9 gene knockout

The CRISPR design tool from the Broad Institute (https://portals.broadinstitute.org/gpp/public/analysis-tools/sgrna-design) was used to design PHB sgRNAs. Oligonucleotides were annealed and cloned into LentiGuide-puro (Addgene) plasmid. For knockout studies, GSCs were transduced with LentiCas9-blast (Addgene) lentiviral construct and selected by blasticidin (10 μg/ml) to obtain GSCs stably expressing Cas9. After that, the GSCs-Cas9 were transduced with PHB sgRNAs lentiviral constructs and selected by puromycin (2 μg/ml). Cell pools were harvested to confirm the knockout efficiency of PHB by IB.

### Inducible KD

GSCs expressing PHB DOX-induced shRNAs were cultured with 100 ng/ml DOX for 4 days before cell lysates were collected to confirm the KD efficiency of PHB. For in vivo inducible KD, GSCs expressing DOX-induced PHB shRNAs were intracranially transplanted into nude mice, supplied with water containing 2 mg/ml DOX. The growth of orthotopic GBM tumors was monitored by bioluminescence imaging using the Calibar IVIS^®^ Spectrum (PerkinElmer) in vivo imaging system.

### Cell viability assays

2000 cells were seeded in 96-well plates with 200 μl culture medium. Cell viability was measured using CellTiter-Glo (Promega, Madison) after the indicated number of days. All data were performed in triplicate.

### Immunoprecipitation and pull-down assay

Cells were collected and lysed in NP-40 lysis buffer (Boster biological technology, AR0107) supplemented with protease inhibitors, incubated on ice for 30 min, and followed by centrifugation at 15,000×*g* for 15 min at 4 °C. The supernatant was subjected to immunoprecipitation with the primary antibody (5 μg of antibody, or normal rabbit or mouse IgG) overnight at 4 °C. The precipitants were extensively washed six times with lysis buffer, boiled with SDS loading buffer and subjected to SDS–PAGE. For the mapping experiments, HEK293 cells were co-transfected with HA-PRDX3 and the full-length or truncated mutants of Flag-PHB for 48 h, and then were treated as described above.

RocA (MCE, HY-19356) was conjugated with cyanogen bromide (CNBr)-activated Sepharose 4B (Sangon Biotech, C500099-0010) in coupling buffer (0.1 M NaHCO_3_, 0.5 M NaCl, pH 8.3) at 4 °C overnight. Cells were collected and lysed in RIPA lysis buffer (20 mM Tris–HCl, pH 7.5; 0.5% NP-40; 150 mM NaCl; 10 mM EDTA; 1% Triton X-100; 1% deoxycholate and ddH_2_O) containing complete protease inhibitor cocktail (Roche, 04693132001) for 30 min, followed by centrifugation at 21,000 × *g* for 20 min at 4 °C. The supernatants were incubated with RocA-conjugated Sepharose 4B at 4 °C overnight. The precipitants were extensively washed six times with lysis buffer, boiled with SDS loading buffer and subjected to SDS–PAGE.

### GST-pull down assay

GST-vector and GST-PRDX3 were expressed in BL21 Competent *E. coli*, purified and incubated with Glutathione Sepharose 4B beads (GE Healthcare, 17-0756-01) according to the manufacturer’s protocol (Amersham Biosciences). For binding assay, Flag-PHB was transfected into HEK293 cells and then was lysed with NP-40 lysis buffer. The cell lysate supernatant was subjected to GST-beads coupled PRDX3 overnight at 4 °C in NP-40 lysis buffer. The precipitants were extensively washed five times with lysis buffer, boiled with SDS-loading buffer and subjected to SDS–PAGE.

### RNA isolation and real-time PCR

Cell pellets were collected and the total RNA was extracted using RNeasy kit (QIAGEN), then reversely transcribed to cDNA with PrimeScript^TM^RT Master Mix (Takara Bio Inc.) according to the manufacturer’s instructions. Real-time PCR was performed with SYBR Green Master Mix (Applied Biosystems) on a cycler (Applied Biosystems). GAPDH or Actin was used for normalization. For miRNA-27a detection, all the Bulge-Loop RT primers for both miRNA-27a and U6 were purchased from RiboBio Co., Ltd. (Guangzhou, China). The relative miRNA levels were normalized to small nuclear RNA U6. The primer pairs used to detect the mRNA and miRNA levels are listed in Supplementary Table 3.

### Oligonucleotides and cell transfection

The *Homo sapiens* (hsa) miRNA-27a mimics (miR-27a sense oligonucleotides), miR-27a inhibitor (miR-27a anti-sense oligonucleotides), and the controls were purchased from RiboBio Co, Ltd. (Guangzhou, China). The mimic and inhibitor control were scrambled oligonucleotide that not produce any identifiable effects on known miRNA function. Cells were transfected with the oligonucleotide using Lipofectamine 3000 (Thermo Fisher Scientific, L3000015) according to the manufacturer’s instruction.

### 3′-UTR-luciferase reporter assay

The wild type and mutant 3′-UTR regions of *PHB* containing predicted miR-27a target site were synthesized (Tsingke Biotechnology Co., Ltd.) and were cloned into the pGL3 vector (Promega, E1741) immediately downstream of the stop codon of the luciferase gene. Either the wild-type or mutant *PHB* 3′-UTR-luciferase was co-transfected with miR-27a mimic/inhibitor in GSCs using Lipofectamine. PRL-TK internal control vector (Promega, E2241) was co-transfected as the endogenous control for luciferase activity. After 48 h, luciferase activity was measured using Dual-Luciferase Reporter Assay (Promega, E1910). The luciferase activity was normalized to PRL-TK activity.

### Determination content of GSH

GSCs were treated with or without 50 μM l-BSO (MedChemExpress, HY-106376A) for 48 h before collection. GSH detection was performed according to the manufacturer’s instructions (Beyotime Biotechnology, S0053). Briefly, cells were washed with 1× PBS and collected, re-suspended with three times the volume of protein removal reagent M solution. Cell samples were subjected to two rapid freeze–thaw cycles using liquid nitrogen and 37 °C water bath. Corresponding detection reagents were added to an appropriate amount of cell samples. After 25 min, GSH was detected by a microplate analyzer at an absorbance of 412 nm. Then GSH content was calculated according to the standard curve.

### Determination activity of catalase

GSCs were treated with or without 50 μM EGCG (MedChemExpress, HY-13653) for 24 h before collection. The determination activity of catalase was performed according to the manufacturer’s instructions (Beyotime Biotechnology, S0051). Briefly, cells were lysed in lysis buffer (Tris–HCl 20 mM PH 7.4, 0.5% NP-40, 250 mM NaCl, 3 mM EGTA, 3 mM EDTA) supplemented with protease inhibitors (Roche) and incubated on ice for 30 min. Cell lysis samples were mixed with the corresponding test solution. After 20 min, catalase activity was detected by a microplate analyzer at an absorbance of 240 nm.

### In vitro limiting dilution assay

For in vitro LDA, decreasing numbers of cells per well (200, 150, 100, 50, and 20) were plated in 96-well plates with 12 replicates for each cell number. Ten days after plating, the presence and number of neurospheres in each well were quantified. Extremely limiting dilution analysis was performed using software available at http://bioinf.wehi.edu.au/software/elda.

### Establishment of GSC-derived intracranial GBM xenografts and combination treatment

Orthotopic GBM xenografts were established through intracranial transplantation of GSCs^32,33^. Briefly, mice used in the studies were 4-week old, female, nu/nu nude mice. GSCs (5 × 10^4^, or indicated number) with indicated treatment were implanted into the right frontal lobe of mice. Mice in each group were treated with one of the following: vehicle control, DOX (1 mg/ml, daily in water) or RocA (2.5 mg/kg, i.p., every 3 days, 6–8 times in total), Irradiation (3 Gy, once a week, four times in total)/TMZ (60 mg/kg, i.p., every 3 days, six times in total), or the combination of DOX/RocA and IR/TMZ starting on the indicated days after tumor implantation. IR was performed with a γ-ray irradiator (Co-60). Xenograft growth was monitored by bioluminescent imaging using the Calibar IVIS^®^ Spectrum (PerkinElmer) in vivo-imaging system every week.

### Measurements of mitochondrial ATP and oxygen consumption

For mitochondrial ATP and oxygen consumption measurement, GSCs were seeded in the XF cell culture microplate (Agilent), intact cellular oxygen consumption was performed using a Seahorse XF96 analyzer. Cells were sequentially challenged with oligomycin (1 μM), carbonyl cyanide 4-(trifluoromethoxy) phenylhydrazone (FCCP) (1 μM), and rotenone plus antimycin A (1 μM).

### Intracellular and mitochondrial ROS quantification

Cells were harvested and trypsinized as a single cell, washed with PBS, re-suspended in 10 μM CM-H2DCFDA (Thermo Fisher Scientific, c6827) in PBS and stained for 15 min at 37 °C in the incubator. After staining, cells were washed twice with PBS and then re-suspended in PBS. H2DCFDA fluoresce signaling was detected by flow cytometer (BD Aria III) at 488 nm channel, and totally 10,000 cells were analyzed per sample. Data analysis was performed with FlowJo 7.6 software.

### Apoptosis assay

After indicated treatments, cells were harvested and trypsinized as single cell. Cells were re-suspended in 100 μl AnnexinV-FITC/PI buffer (Promega) and incubated for 15 min at room temperature in dark. The rate of apoptosis was measured by a flow cytometer (BD Aria III). Totally 10,000 cells were analyzed per sample. Data analysis was performed with FlowJo 7.6 software.

### Ubiquitination

GSCs were treated with 10 μM MG132 for 12 h before collection. Cells were lysed in NP-40 lysis buffer (Boster biological technology, AR0107) supplemented with protease inhibitors (Roche) and N-Ethylmaleimide (Sigma, E3876), incubated on ice for 30 min, and followed by centrifugation at 15,000 × *g* for 15 min at 4 °C. The supernatant was subjected to immunoprecipitation with anti-Flag M2 affinity gel (Sigma, A2220) or protein G sepharose (GE Healthcare, 17-0618-01). The immunoprecipitants were detected by immunoblotting with anti-ubiquitin antibody, anti-ubiquitin Lys48-specific antibody, or anti-Flag antibody to detect ubiquitination of PRDX3.

### RNA sequencing

Total RNA was isolated from cells using the RNeasy Mini Kit (QIAGEN). Strand-specific cDNA libraries were generated using the Illumina TruSeq Stranded Total RNA Library Prep Kit with Ribo-Zero Gold. cDNA quality was determined using the Agilent high-sensitivity DNA kit on an Agilent 2100 BioAnalyzer (Agilent Technologies). Paired-end 125 bp reads were generated on an Illumina HiSeq 2500 instrument at the Oebiotech.corp. Reads were aligned to the GRCh38.p7 genome using TopHat v2.1.1 with the library type option set to first strand. Fragments per kilobase per millions (FPKMs) of known genes were calculated using eXpress v1.5.1.

### PDX models

PDXs were established through subcutaneous transplantation of GBM patient cells into NOD/SCID mice. Mice used in the studies were 4-week old, female. After mass formation, mice were randomly divided into four groups, treated with one of the followings: vehicle control, RocA (2.5 mg/kg, i.p., every 3 days, eight times), IR (3 Gy, once a week, four times), or the combination of RocA and IR starting on the indicated days after tumor implantation. IR was performed with a γ-ray irradiator (Co-60). Tumor size was evaluated every 2 days by caliper measurements and the approximate volume of the mass was calculated using the formula [(small diameter)^2^ × (large diameter)×0.5]. Once tumor volume exceeded 1500 mm^3^, mice were sacrificed.

### Liquid chromatography-tandem mass spectrometry analysis of Rocaglamide

The smashed mouse brains and extracted serums were added with 80% mixture of methanol and acetonitrile (1:1), pre-chilled at −80 °C and incubated at −80 °C for 1 h. Then, the samples were centrifuged at 18,000×*g* for 10 min and the supernatant was dried with SpeedVac. The pellets were rediscounted with 100 μl of 50% methanol solution and centrifuged at 18,000×*g* for 10 min at 4 °C for supernatants collection. Extracted samples and standards were analyzed by Triple Quad 6500 mass spectrometer (SCIEX) coupled with the UltiMate 3000 HPLC system (Thermo Fisher Scientific). The LC separation was performed on an ACQUITY UPLC HSS T3 column (100 mm × 2.1 mm) (WATERS). For Rocaglamide multiple reaction monitoring (MRM) detection, 506.0/352.1 transition was chosen in positive ion mode. Peak integration and statistical analyses were performed using MultiquantTM 2.1 software (SCIEX).

### Microarray analysis from GEO database

Microarray data of GEO (http://www.ncbi.nlm.nih.gov/geo) GSE86237 and GSE54791, were enrolled in the study. GSE86237 profile included microarray data from 19 panels of GSCs and 7 panels of BTCs both isolated from primary GBM samples^36^. GSE54791 database contained gene-profiling data from three pairs of GSCs and NSTCs^37^.

### Statistics and reproducibility

All grouped data are presented as mean ± SD or SEM from studies performed at least in triplicate unless otherwise specified. For bar graphs, the unpaired two-sided Student’s *t-*test or Welch’s two-sided *t-*test was used for the comparison between unpaired two groups. Two-way ANOVA was applied for multi-group data comparison. A probability value <0.05 was considered significant. For the survival analysis, Kaplan–Meier survival curves were analyzed by using Log-rank Mantel–Cox test comparing the different patient or mouse groups. IC_50_ of RocA was calculated using nonlinear regression analyses based on dose–response curves. For all figures presented in box-and-whisker format, the center line represents the median and the lower and upper limits of the box represent the 25th and 75th percentiles. The maximum and minimum are connected to the center box through the vertical lines (whiskers). GraphPad Prism Software 8.0 (GraphPad Software, Inc.) and Microsoft Office Excel (office 2013) were used for all statistical analyses.

Results in Figs. 1a–c, g, h, 2a, b, 4a–d, f, g (left and right panels), h, 5i, 6a–c; Supplementary Figs. 1c, g, h, 2h, 3g, 4a, b, 5c, g, 5h, i (right panels), k, p, 6f, 6i, j, 7g–j, 9c. are representative data of three independent repeats. And there were similar results in three independent repeats.

### Reporting summary

Further information on research design is available in the Nature Research Reporting Summary linked to this article.

## Supplementary information

Supplementary Information

Reporting Summary

## Data Availability

The microarray data referenced during the study are available from the Gene Expression Omnibus under accession numbers GSE86237 and GSE54791. The RNA sequencing data for control GSCs and *PHB* KO GSCs have been deposited in the public database under the accession code HRA000843. The expression of PHB and the pathological characteristics of human glioma patients of the tissue microarray is provided in Supplementary Table 1. The sgRNAs and shRNAs used in this study are provided in Supplementary Table 2. The primer sequences used for Q-PCR are provided in Supplementary Table 3. Source data are provided with this paper. The remaining data are available within the Article, Supplementary Information and Source Data. Source data are provided with this paper.

## Source data

Source Data
