# Supplementary material for: Suppression of mitochondrial ROS by prohibitin drives glioblastoma progression and therapeutic resistance
Source: Nat Commun. 2021 Jun 17;12:3720. doi: 10.1038/s41467-021-24108-6 (PMC8211793; doi:10.1038/s41467-021-24108-6)
Supplement: Supplementary file 1 — Supplementary Information [file 41467_2021_24108_MOESM1_ESM.pdf]

Supplementary Information

Suppression of Mitochondrial ROS by Prohibitin Drives Glioblastoma Progression and Therapeutic Resistance

Haohao Huang[1,6,7], Songyang Zhang[1,7], Yuanyuan Li[1,7], Zhaodan Liu[1], Lanjuan Mi[1], Yan Cai[1], Xinzheng Wang[1], Lishu Chen[1], Haowen Ran[1], Dake Xiao[1], Fangye Li[5], Jiaqi Wu[1], Tingting Li[1], Qiuying Han[1], Liang Chen[1], Xin Pan[1], Huiyan Li[1], Tao Li[1], Kun He[1], Ailing Li[1,4], Xuemin Zhang[1,3,4], Tao Zhou[1,2], Qing Xia[1]* and Jianghong Man[1,2]*

Affiliations

[1] State Key Laboratory of Proteomics, National Center of Biomedical Analysis, Beijing 100850, China

[2] Nanhu Laboratory, Jiaxing, Zhejiang Province 314002, China.

[3] State Key Laboratory of Toxicology and Medical Countermeasures, Beijing Institute of Pharmacology and Toxicology, National Center of Biomedical Analysis, Beijing 100850, China

[4] The First Hospital of Jilin University, Changchun 130021, China

[5] Department of Neurosurgery, First Medical Center of PLA General Hospital, Beijing 100853, China

[6] Department of Neurosurgery, General Hospital of Central Theater Command of Chinese People's Liberation Army, Wuhan 430070, PR China.

[7] These authors contributed equally: Haohao Huang, Songyang Zhang, Yuanyuan Li

*Correspondence: qxia@ncba.ac.cn (Q.X.), jhman@ncba.ac.cn (J.H.M.)

Supplementary Figures 1 – 9

Supplementary Tables 1 – 3

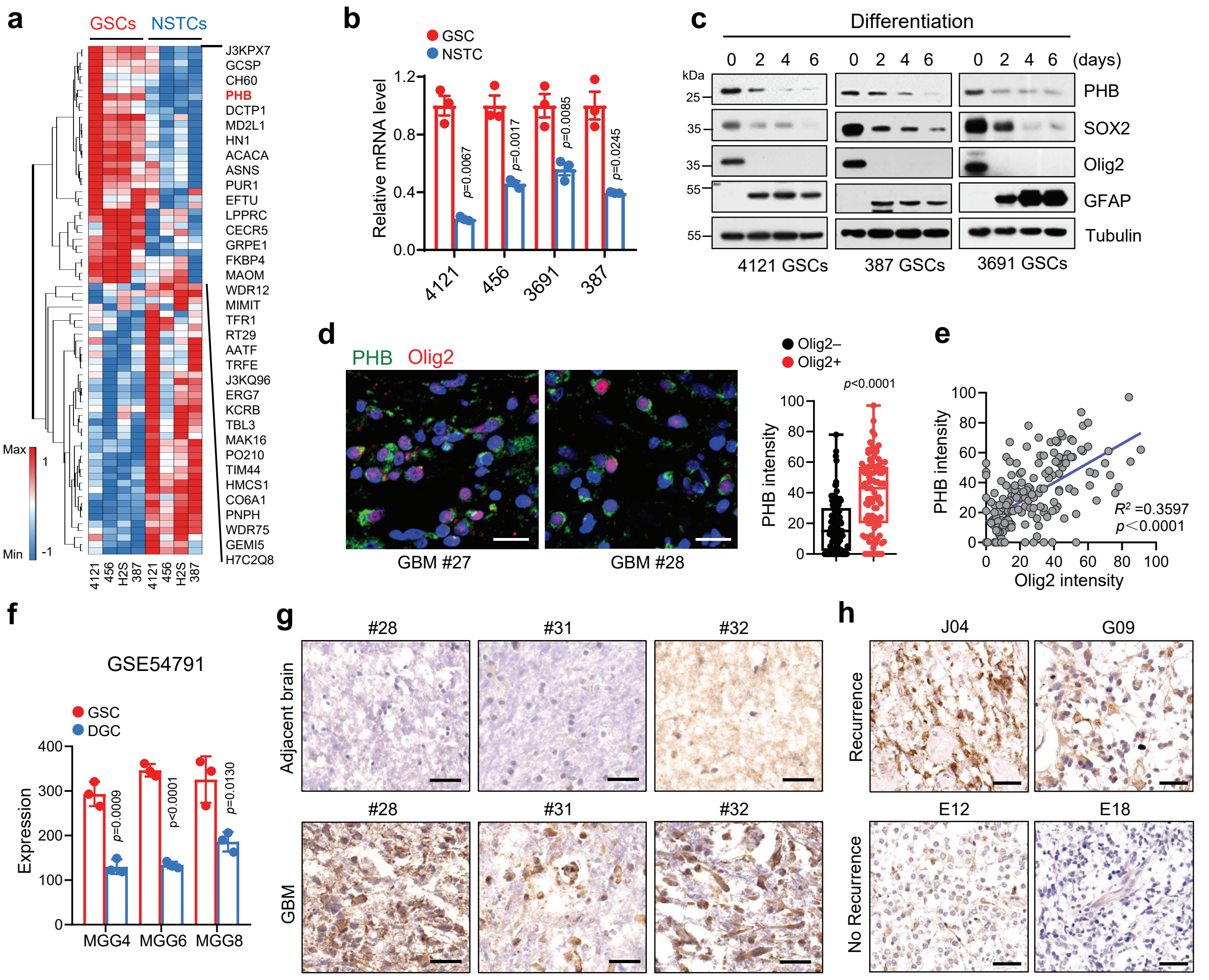

Supplementary Fig. 1 | PHB is highly expressed in GSCs. a, Heatmap representation of upregulated and downregulated proteins (the average change > 2 fold) screened by mass spectrometry in four GSCs compared to the matched NSTCs derived from human GBM xenografts. Raw data were $\log_2$ transformed. A relative color scheme used the minimum and maximum values in each row to convert values to colors. Red is high expression and blue is low. **b,** Q-PCR analysis of PHB mRNA levels in multiple GSCs and matched NSTCs are shown. (Mean ± SD, n=3, biologically independent experiments, Welch's two-sided *t*-test). **c,** IB analysis of PHB, SOX2, Olig2 and GFAP during GSC differentiation on the indicated days. GSC differentiation was induced by serum (10% FBS). **d,e,** Representative IF images of human primary GBM specimens stained with anti-PHB (green) and anti-Olig2 (red). Nuclei were counterstained with Hoechst (blue) (**d**, left). Scale bars, 40μm. Quantifications of PHB staining intensity in Olig2+ (n=111) and Olig2- (n=98) cells (5 random microscope fields from 3 tumors) are shown (**d**, right). (Boxplots represent the median, 25th and 75th percentiles. The maximum and minimum are connected to the center box through the vertical lines (whiskers). Welch's two-sided *t*-test). Pearson correlation between PHB and Olig2 staining intensity in GBM cells are shown (**e**). **f,** mRNA expression of PHB in MGG4, MGG6 and MGG8 GSCs relative to the matched differentiated glioma cells (DGCs) from GEO database (GSE54791). (Mean ± SD, n=3, biologically independent experiments, Unpaired two-sided Student's *t*-test). **g,** IHC analysis of PHB in primary GBMs and matched adjacent brain tissues are shown. Scale bars, 50 μm. **h,** Representative images of PHB IHC staining in recurrent gliomas (I09, J04, G09, Grade III) and non-recurrent gliomas (A13, E12, E18, Grade III) in glioma tissue microarray are shown. Scale bars, 50 μm.

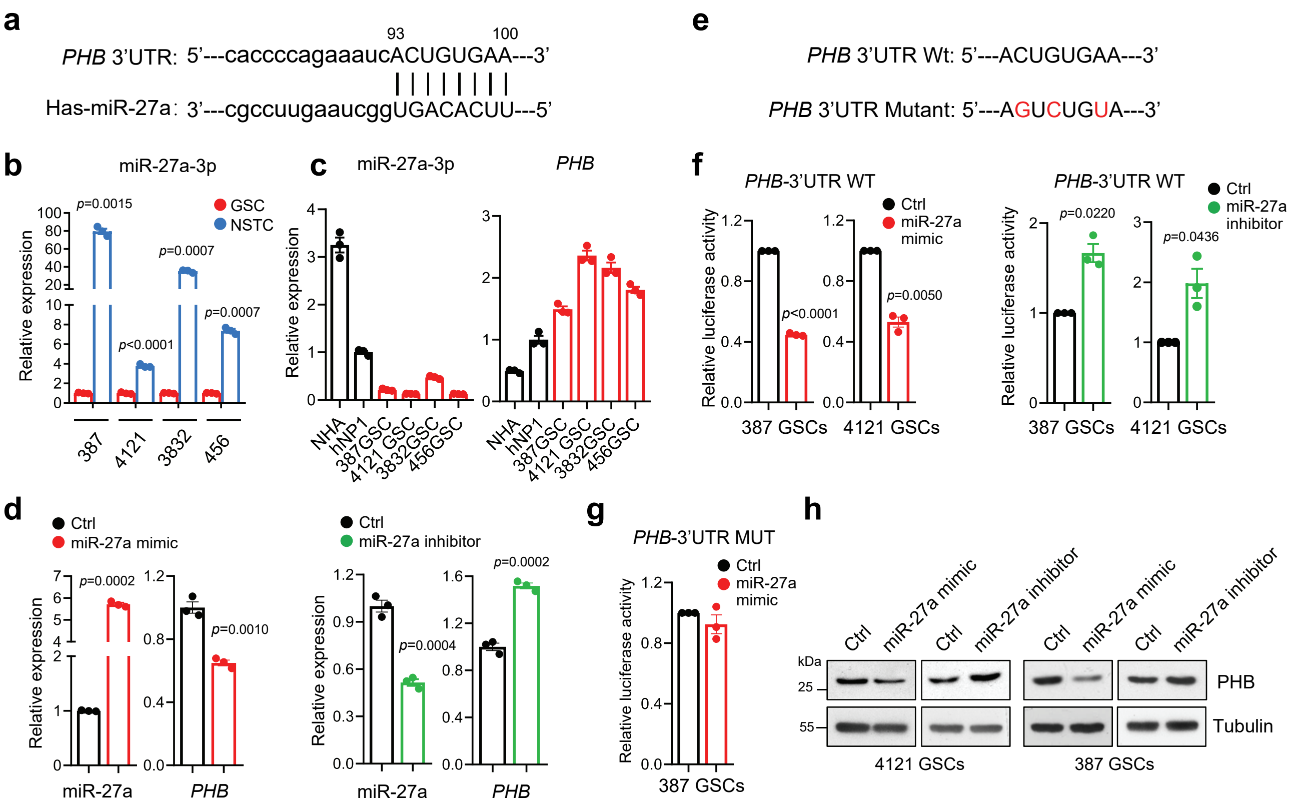

Supplementary Fig. 2 | miRNA27a mediated the upregulation of PHB in GSCs. a, Alignment of miRNA-27a-3p and its binding site in the 3'UTR of *PHB*. **b,c,** Q-PCR analysis of miRNA-27a-3p levels in multiple GSCs and matched NSTCs (**b**), or in multiple GSCs, NHA and hNP1 (**c**). (Mean±SD, n=3, biologically independent experiments). **d,** Q-PCR analysis of miRNA-27a-3p and *PHB* mRNA levels in 387 GSCs with indicated treatments. (Mean±SD, n=3, biologically independent experiments). **e-g,** The wild-type and a mutated type of binding site of miRNA-27a in 3' UTR of *PHB* are shown (**e**). Luciferase activity of the reporter construct containing the wild-type (**f**) or miR-27a-binding mutant 3' UTR of *PHB* (**g**) was measured after co-transfection of the reporter with miRNA-27a mimic or inhibitor in GSCs. (Mean±SD, n=3, biologically independent experiments). **h,** IB showing levels of indicated proteins in 4121 and 387 GSCs with indicated treatments.

Welch's two-sided *t*-test (**b, d, f, g**).

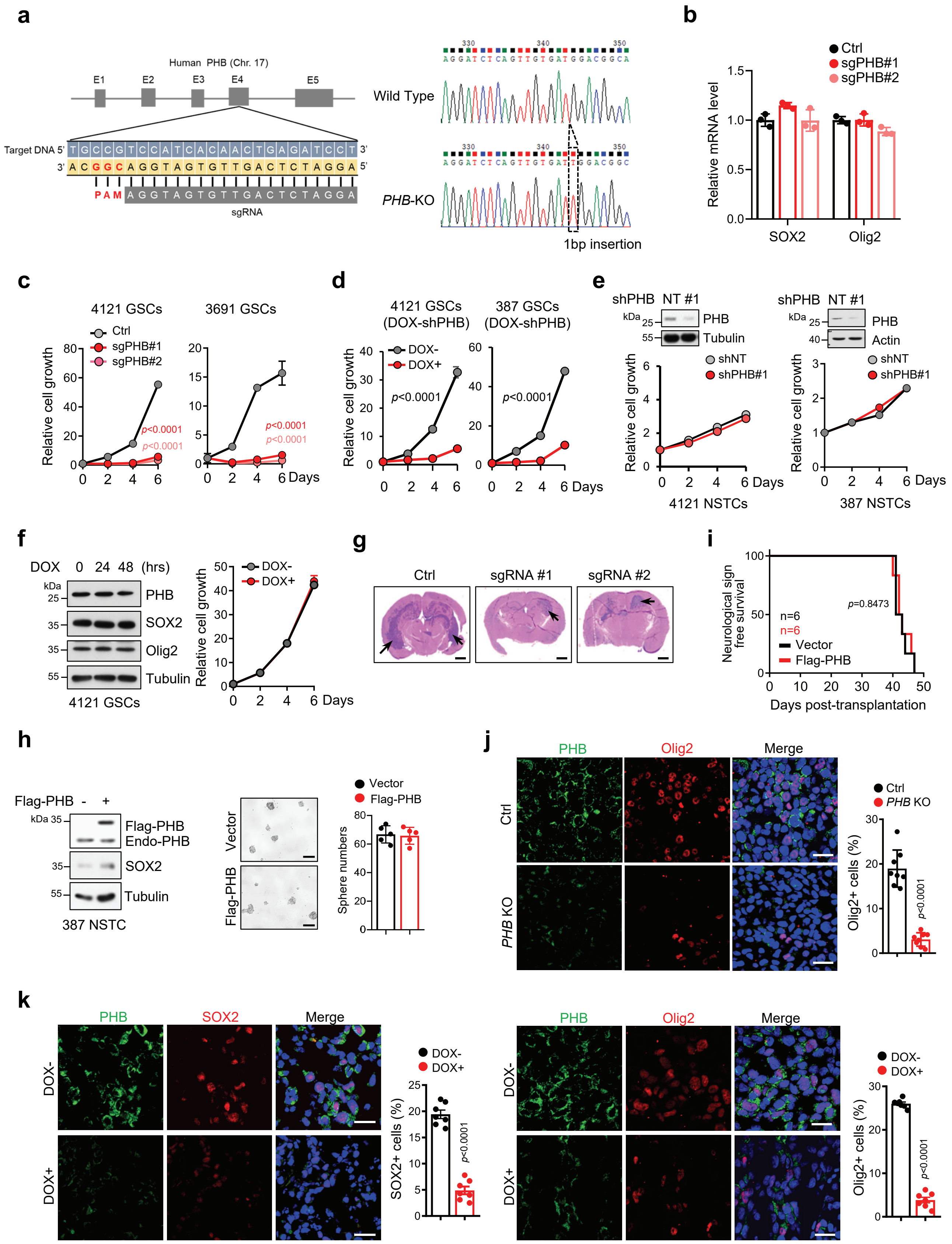
a
Human PHB (Chr. 17)
E1 E2 E3 E4 E5
Target DNA 5' TGCCGTCCATCACAACTGAGATCCT 3'
3' ACGGCAGGTAGTGTTGACTCTAGGA 5'
PAM AGGTAGTGTTGACTCTAGGA
sgRNA
Wild Type
PHB-KO
1bp insertion
b
Ctrl
sgPHB#1
sgPHB#2
Relative mRNA level
SOX2
Olig2
c
4121 GSCs
3691 GSCs
Relative cell growth
p<0.0001
Days
d
4121 GSCs (DOX-shPHB)
387 GSCs (DOX-shPHB)
DOX-
DOX+
p<0.0001
e
shPHB NT #1
PHB
Tubulin
Actin
shNT
shPHB#1
4121 NSTCs
387 NSTCs
f
DOX 0 24 48 (hrs)
PHB
SOX2
Olig2
Tubulin
4121 GSCs
g
Ctrl
sgRNA #1
sgRNA #2
i
Neurological sign free survival
p=0.8473
n=6
n=6
Vector
Flag-PHB
Days post-transplantation
h
Flag-PHB - +
Flag-PHB
Endo-PHB
SOX2
Tubulin
387 NSTC
Vector
Flag-PHB
Sphere numbers
j
PHB
Olig2
Merge
Ctrl
PHB KO
Olig2+ cells (%)
p<0.0001
k
PHB
SOX2
Merge
DOX-
DOX+
SOX2+ cells (%)
p<0.0001
PHB
Olig2
Merge
Olig2+ cells (%)
p<0.0001

Supplementary Fig.3 | PHB promotes GSC self renewal and tumor progression. a, Schematic illustration of the *PHB* locus. The CRISPR target site and PAM motif are indicated. Sanger sequence of *PHB* gene in the control and KO GSCs. A base of nucleic acid (T) was inserted at 344 of PHB coding sequence resulting in frame shift mutation. **b**, Q-PCR analysis of SOX2 and Olig2 mRNA levels in control and *PHB* KO GSCs (4121). (Mean±SD, n=3, biologically independent experiments). **c,d,** *PHB* KO (**c**) or PHB inducible-KD (**d**) inhibited cell growth of GSCs, as assessed by cell viability assay. (Mean±SD, n=3, biologically independent experiments). **e,** PHB knockdown had no obvious effect on cell growth of NSTCs, as measured by cell viability assay. (Mean±SD, n=3, biologically independent experiments). **f,** DOX had no effect on stem cell markers expression nor cell growth in 4121 GSCs. (Mean±SD, n=3, biologically independent experiments). **g,** Ctrl or *PHB* KO GSCs (4121) ($5X10^4$/mouse) were implanted into brains of nude mice (nu/nu). Representative images of H&E stained sections of mouse brains collected on day 30 after GSC transplantation are shown. Scale bars, 1mm. **h,** Overexpression of Flag-PHB had no effect on SOX2 expression or tumor sphere formation in 387 NSTCs. (Mean±SD, n=5, biologically independent experiments). Scale bars, 100μm. **i,** Vector or Flag-PHB expressing 387 NSTCs ($5X10^5$/mouse) were implanted into brains of nude mice (nu/nu). Kaplan-Meier survival plot of mice is shown (Log rank Mantel-Cox test). **j,k,** Co-IF staining of PHB (green) and SOX2 or Olig2 (red) in GBM xenografts derived from Ctrl or *PHB* KO 4121 GSCs (**j**), or control or PHB KD 4121 GSCs (**k**) are shown (left). Quantifications of SOX2+ or Olig2+ cells are shown (right). (Mean±SD, images n=8 (*PHB* KO) or n=7 (PHB KD), from 4 biologically independent samples). Nuclei were counterstained with Hoechst (blue). Scale bars, 20μm.

Two-way ANOVA (**c-f**), Unpaired two-sided Student's *t*-test (**h, k**) or Welch's two-sided *t*-test (**j**).

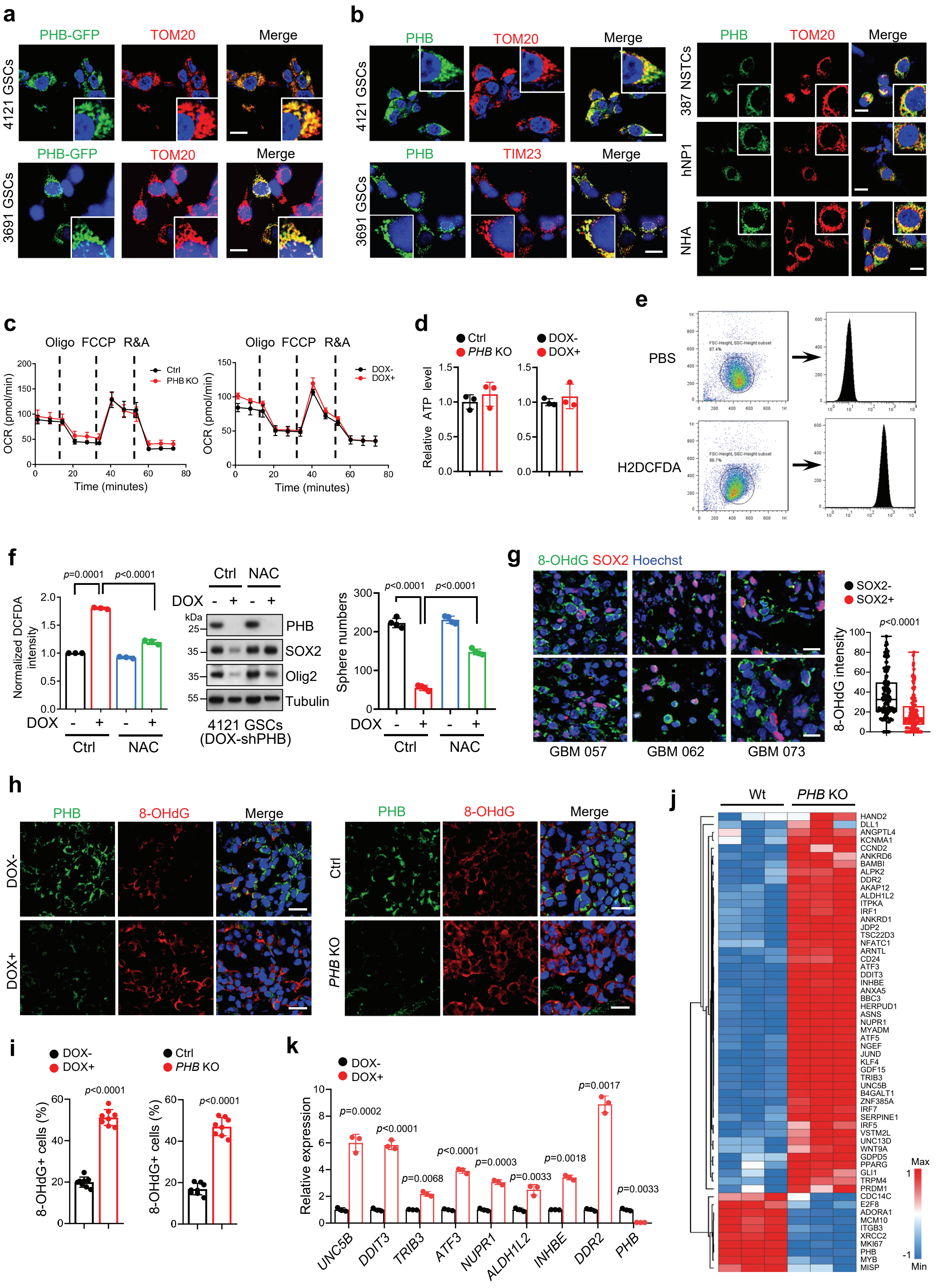

a
PHB-GFP
TOM20
Merge
4121 GSCs
3691 GSCs
b
PHB
TOM20
TIM23
Merge
4121 GSCs
3691 GSCs
387 NSTCs
hNP1
NHA
c
Oligo FCCP R&A
OCR (pmol/min)
Time (minutes)
Ctrl
PHB KO
DOX-
DOX+
d
Ctrl
PHB KO
DOX-
DOX+
Relative ATP level
e
PBS
H2DCFDA
f
Normalized DCFDA intensity
p=0.0001
p<0.0001
DOX
Ctrl
NAC
kDa
PHB
SOX2
Olig2
Tubulin
4121 GSCs (DOX-shPHB)
Sphere numbers
g
8-OHdG SOX2 Hoechst
GBM 057
GBM 062
GBM 073
SOX2-
SOX2+
p<0.0001
8-OHdG intensity
h
PHB
8-OHdG
Merge
DOX-
DOX+
Ctrl
PHB KO
i
DOX-
DOX+
Ctrl
PHB KO
8-OHdG+ cells (%)
p<0.0001
j
Wt
PHB KO
HAND2
DLL1
ANGPTL4
KCNMA1
CCND2
ANKRD6
BAMBI
ALPK2
DDR2
AKAP12
ALDH1L2
ITPKA
IRF1
ANKRD1
JDP2
TSC22D3
NFATC1
ARNTL
CD24
ATF3
DDIT3
INHBE
ANXA5
BBC3
HERPUD1
ASNS
NUPR1
MYADM
ATF5
NGEF
JUND
KLF4
GDF15
TRIB3
UNC5B
B4GALT1
ZNF385A
IRF7
SERPINE1
IRF5
VSTM2L
UNC13D
WNT9A
GDPD5
PPARG
GLI1
TRPM4
PRDM1
CDC14C
E2F8
ADORA1
MCM10
ITGB3
XRCC2
MKI67
PHB
MYB
MISP
Max
Min
k
DOX-
DOX+
Relative expression
p=0.0002
p<0.0001
p=0.0068
p<0.0001
p=0.0003
p=0.0033
p=0.0018
p=0.0017
p=0.0033
UNC5B
DDIT3
TRIB3
ATF3
NUPR1
ALDH1L2
INHBE
DDR2
PHB

Supplementary Fig. 4 | PHB specific mediates low levels of mitochondrial peroxide in GSCs. a, IF staining of TOM20 (red) in GSCs (4121 and 3691) transduced with PHB-GFP are shown. Scale bars, 10μm. **b** Co-IF staining of PHB (green) and TOM20 or TIM23 (red) in GSCs (4121 or 3691), NSTCs (387), hNP1 and NHA are shown. Scale bars, 10μm. **c,** Mitochondrial oxygen consumption rate (OCR) of Ctrl and *PHB* KO 4121 GSCs (left) (Mean$\pm$SD, Ctrl n=3, *PHB* KO n=5, biologically independent experiments), or of DOX-shPHB 4121 GSCs treated with or without DOX (right) (Mean$\pm$SD, DOX- n=4, DOX+ n=6, biologically independent experiments), under basal conditions and in response to oligomycin (1 μM), FCCP (0.5 μM) and rotenone / antimycin A (1 μM). **d,** *PHB* KO or PHB inducible KD had no effect on mitochondrial ATP generation in GSCs (4121). (Mean$\pm$SD, n=3, biologically independent experiments). **e,** Gating strategy to determine the intracellular ROS level by H2DCFDA staining. **f,** Ctrl or PHB KD GSCs (4121) were treated with vehicle control or NAC (5mM) for 36 hours. Flow cytometry analysis of peroxide by DCFDA staining are shown (left). IB of PHB, SOX2 and Olig2 levels are shown (middle). Quantifications of tumor sphere numbers (2,000 cells / well) formed by GSCs are shown (right). (Mean$\pm$SD, n=3, biologically independent experiments). **g,** Co-IF staining of 8-OHdG (green) and SOX2 (red) in primary GBM specimens are shown. Quantifications of 8-OHdG staining intensity in SOX2+ (n=123) and SOX2- (n=128) cells are shown (right). (Boxplots represent the median, 25th and 75th percentiles. The maximum and minimum are connected to the center box through the vertical lines (whiskers)). Nuclei were counterstained with Hoechst (blue). Scale bars, 20μm. **h,i,** Co-IF staining of PHB (green) and 8-OHdG (red) in GBM xenografts derived from Ctrl or *PHB* KO GSCs (4121) (**h**, right), or PHB inducible-KD GSCs (4121) (**h**, left) are shown. Quantifications of 8-OHdG+ cells are shown (**i**). (Mean$\pm$SD, images n=8, from 4 biologically independent samples). Nuclei were counterstained with Hoechst (blue). Scale bars, 40μm. **j,** RNA-seq analysis in control and *PHB* KO 4121 GSCs. Heat map of selected genes related to regulation of cell death, cell differentiation and cell cycle is shown. **k**, Q-PCR analysis of mRNA levels of indicated genes in control and PHB inducible-KD 4121 GSCs. (Mean$\pm$SD, n=3, biologically independent experiments).

Unpaired two-sided Student's *t*-test (**d, i**) or Welch's two-sided *t*-test (**f, g, k**).

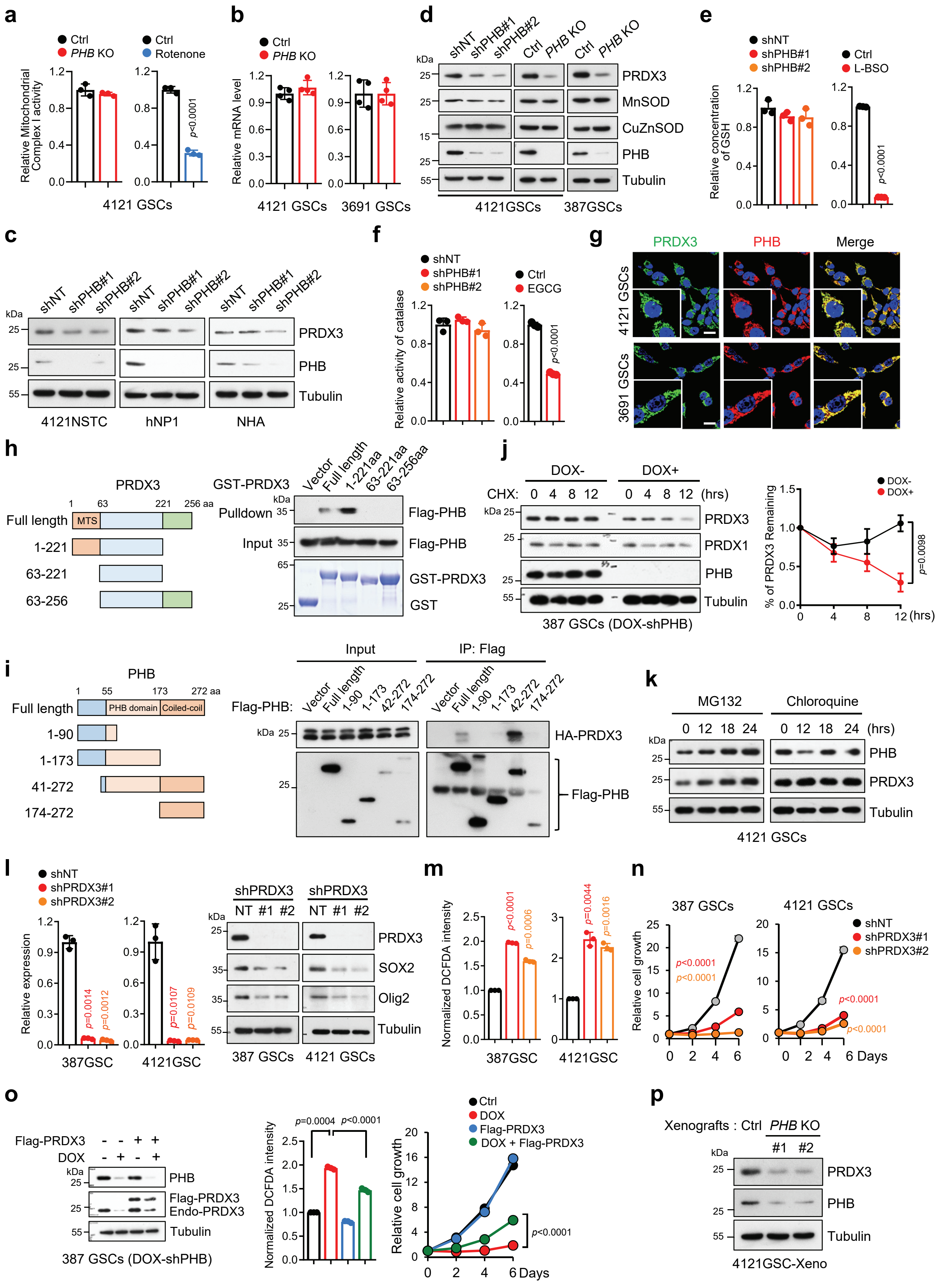

a
Ctrl
PHB KO
Ctrl
Rotenone
Relative Mitochondrial Complex I activity
p<0.0001
4121 GSCs
b
Ctrl
PHB KO
Relative mRNA level
4121 GSCs
3691 GSCs
c
shNT
shPHB#1
shPHB#2
PRDX3
PHB
Tubulin
4121NSTC
hNP1
NHA
d
Ctrl
PHB KO
PRDX3
MnSOD
CuZnSOD
PHB
Tubulin
4121GSCs
387GSCs
e
shNT
shPHB#1
shPHB#2
Ctrl
L-BSO
Relative concentration of GSH
p<0.0001
f
shNT
shPHB#1
shPHB#2
Ctrl
EGCG
Relative activity of catalase
p<0.0001
g
PRDX3
PHB
Merge
4121 GSCs
3691 GSCs
h
PRDX3
Full length
MTS
1-221
63-221
63-256
GST-PRDX3
Vector
Full length
1-221aa
63-221aa
63-256aa
Pulldown
Input
Flag-PHB
GST-PRDX3
GST
j
DOX-
DOX+
CHX: 0 4 8 12 (hrs)
PRDX3
PRDX1
PHB
Tubulin
387 GSCs (DOX-shPHB)
% of PRDX3 Remaining
p=0.0098
(hrs)
i
PHB
Full length
PHB domain
Coiled-coil
1-90
1-173
41-272
174-272
Input
IP: Flag
Flag-PHB:
Vector
Full length
1-90
1-173
42-272
174-272
HA-PRDX3
Flag-PHB
k
MG132
Chloroquine
0 12 18 24 (hrs)
PHB
PRDX3
Tubulin
4121 GSCs
l
shNT
shPRDX3#1
shPRDX3#2
Relative expression
p=0.0014
p=0.0012
p=0.0107
p=0.0109
387GSC
4121GSC
shPRDX3
NT #1 #2
PRDX3
SOX2
Olig2
Tubulin
387 GSCs
4121 GSCs
m
Normalized DCFDA intensity
p<0.0001
p=0.0006
p=0.0044
p=0.0016
387GSC
4121GSC
n
387 GSCs
4121 GSCs
shNT
shPRDX3#1
shPRDX3#2
Relative cell growth
p<0.0001
p<0.0001
p<0.0001
p<0.0001
Days
o
Flag-PRDX3
DOX
PHB
Flag-PRDX3
Endo-PRDX3
Tubulin
387 GSCs (DOX-shPHB)
Normalized DCFDA intensity
p=0.0004
p<0.0001
Ctrl
DOX
Flag-PRDX3
DOX + Flag-PRDX3
Relative cell growth
p<0.0001
Days
p
Xenografts : Ctrl
PHB KO
#1 #2
PRDX3
PHB
Tubulin
4121GSC-Xeno

Supplementary Fig. 5 | PHB associates with and stabilizes PRDX3 by inhibiting its ubiquitin-proteasome degradation. **a**, Mitochondrial complex I activity of *PHB* KO GSCs was calculated and normalized to control GSCs by measuring NADH oxidation rate. Rotenone (1μM) was used as a positive control. (Mean±SD, n=3, biologically independent experiments). **b,** Q-PCR analysis showing the relative mRNA levels of PRDX3 in Ctrl and *PHB* KO GSCs. (Mean±SD, n=4, biologically independent experiments). **c,** IB showing levels of PRDX3 in PHB KD 4121 NSTCs, hNP1 and NHA. **d,** IB showing levels of indicated proteins in 4121 and 387 GSCs with PHB KO or PHB KD. **e,** The activity of GSH is examined in control and PHB KD 4121 GSCs. L-buthionine sulfoximine (l-BSO) was used as a positive control (50μM, 48 hours). (Mean±SD, n=3, biologically independent experiments). **f,** The activity of catalase is examined in control and PHB KD GSCs. Epigallocatechin gallate (EGCG) was used as a positive control (50μM, 24 hours). (Mean±SD, n=3, biologically independent experiments). **g,** Co-IF staining of PRDX3 (green) and PHB (red) in GSCs are shown. Scale bars, 10μm. **h,** Schematic drawing of the domains of PRDX3 (left). The PHB-PRDX3 interaction was analyzed by the GST-pull down assay with GST or GST-fused PRDX3 (or its truncated mutants) and cell lysates from HEK293 cells expressing Flag-PHB. **i,** Schematic drawing of the domains of PHB (left). The PHB-PRDX3 interaction was analyzed by Co-IP with anti-flag M2 beads in HEK293 cells expressing HA-PRDX3 and Flag-tagged full length or truncated PHB (right). **j,** IB showing the levels of PRDX3 and PHB in GSCs treated with MG132 (10μM) or Chloroquine (20μM) for indicated time. (Mean±SD, n=3, biologically independent experiments). **k,** IB showing the CHX (50μg/ml) chase analysis of PRDX3 protein degradation at indicated time points in GSCs with or without PHB inducible-KD. Quantifications of relative protein levels of PRDX3 are shown (right). **l-n,** Knockdown of PRDX3 increased peroxide levels as indicated by DCFDA fluorescence, were measured by flow cytometry (**m**), and inhibited GSC cell growth as assessed by cell viability assay (**n**). (Mean±SD, n=3 (**l, m**), n=4 (**n**), biologically independent experiments). The knockdown efficiency of PRDX3 were assessed by Q-PCR analysis (**l**, left) and IB (**l**, right). (Mean±SD, n=3, biologically independent experiments). **o,** Ectopic expression of Flag-PRDX3 rescued the induction of peroxide levels and the inhibition of cell growth by PHB depletion in GSCs. IB showing the levels of PRDX3 and PHB in GSCs (left). The peroxide levels, as indicated by DCFDA fluorescence, were measured by flow cytometry (middle). Cell growth of GSCs were assessed by cell viability assay (right). (Mean±SD, n=4, biologically independent experiments). **p,** *PHB* KO resulted in a decrease of PRDX3 protein levels in 4121 GSCs-derived xenografts.

Data are represented as mean ± SD. Unpaired two-sided Student's *t*-test (**a, b, e, f**), Welch's two-sided *t*-test (**l, m, o** left), Two-way ANOVA (**j, n, o** right).

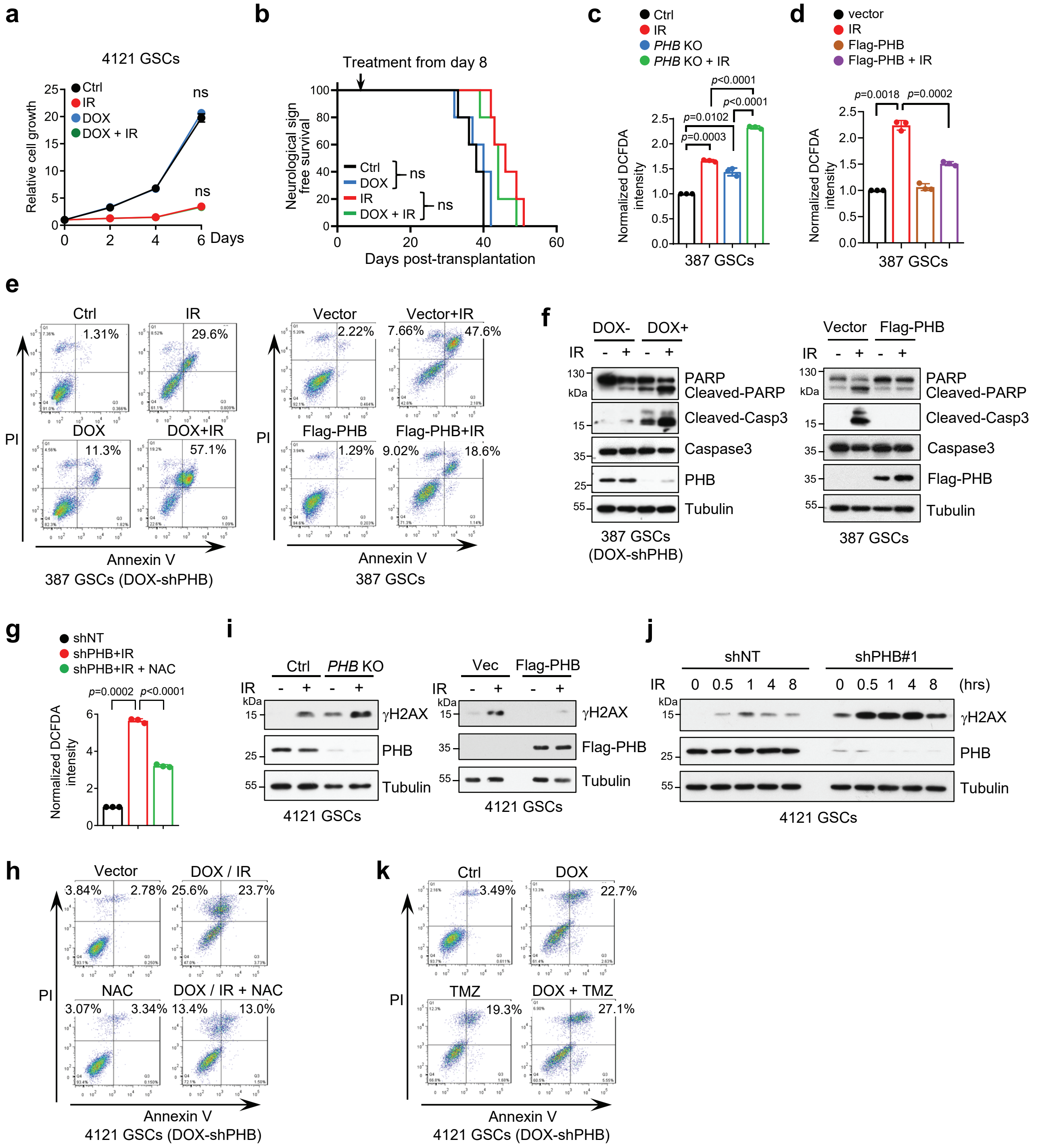

Supplementary Fig. 6 | PHB promotes GSC radio-resistance. a,b, DOX treatment (100ng/ml) has no effect on growth of GSCs, nor the sensitivity of GSCs to IR. Cell growth were assessed by cell viability assay at indicated time points (**a**). (Mean±SD, n=3, biologically independent experiments, Two-way ANOVA). Nude mice (nu/nu) bearing 4121 GSC-derived orthotopic xenografts were randomly grouped (n=5 for each group) at day 8 after implantation and treated with control, DOX (2mg/ml in drinking water), IR (3Gy, once a week, 4 times) or the combined treatment. Kaplan-Meier survival plot of mice is shown (**b**). (Log rank Mantel-Cox test). **c,d,** The peroxide levels, as indicated by DCFDA fluorescence, were measured by flow cytometry in 387 GSCs with indicated treatments. IR, 3Gy for 48 hours (**c**) or 72 hours (**d**). (Mean ± SD, n=3, biologically independent experiments, Unpaired two-sided Student's *t*-test). **e,** Cell apoptosis were measured by flow cytometry in GSCs with indicated treatments. IR, 3Gy for 48 hours (left) or 72 hours (right).

f, IB showing levels of cleaved-PARP, cleaved-caspaspe3, caspase3 and PHB in GSCs with indicated treatments. IR, 3Gy for 48 hours (left) or 72 hours (right). **g,h,** NAC (5mM) treatment rescued the induction of peroxide (**g**) and increase of cell apoptosis (**h**) by combined PHB inducible-KD and IR treatment in 4121 GSCs. IR, 3Gy for 24 hours. (Mean$\pm$SD, n=3, biologically independent experiments, Welch's two-sided *t*-test). **i,j** IB showing levels of γH2AX in control and *PHB* KO GSCs, and vector or Flag-PHB expressing GSCs treated with or without IR for 1 hour (**i**), or in control and PHB KD GSCs treated with IR for indicated time points (**j**). IR, 3Gy. **k,** Cell apoptosis were measured by flow cytometry in 4121 GSCs (DOX-shPHB) with indicated treatments. IR, 3Gy for 48 hours. TMZ, 300 μM for 48 hours.

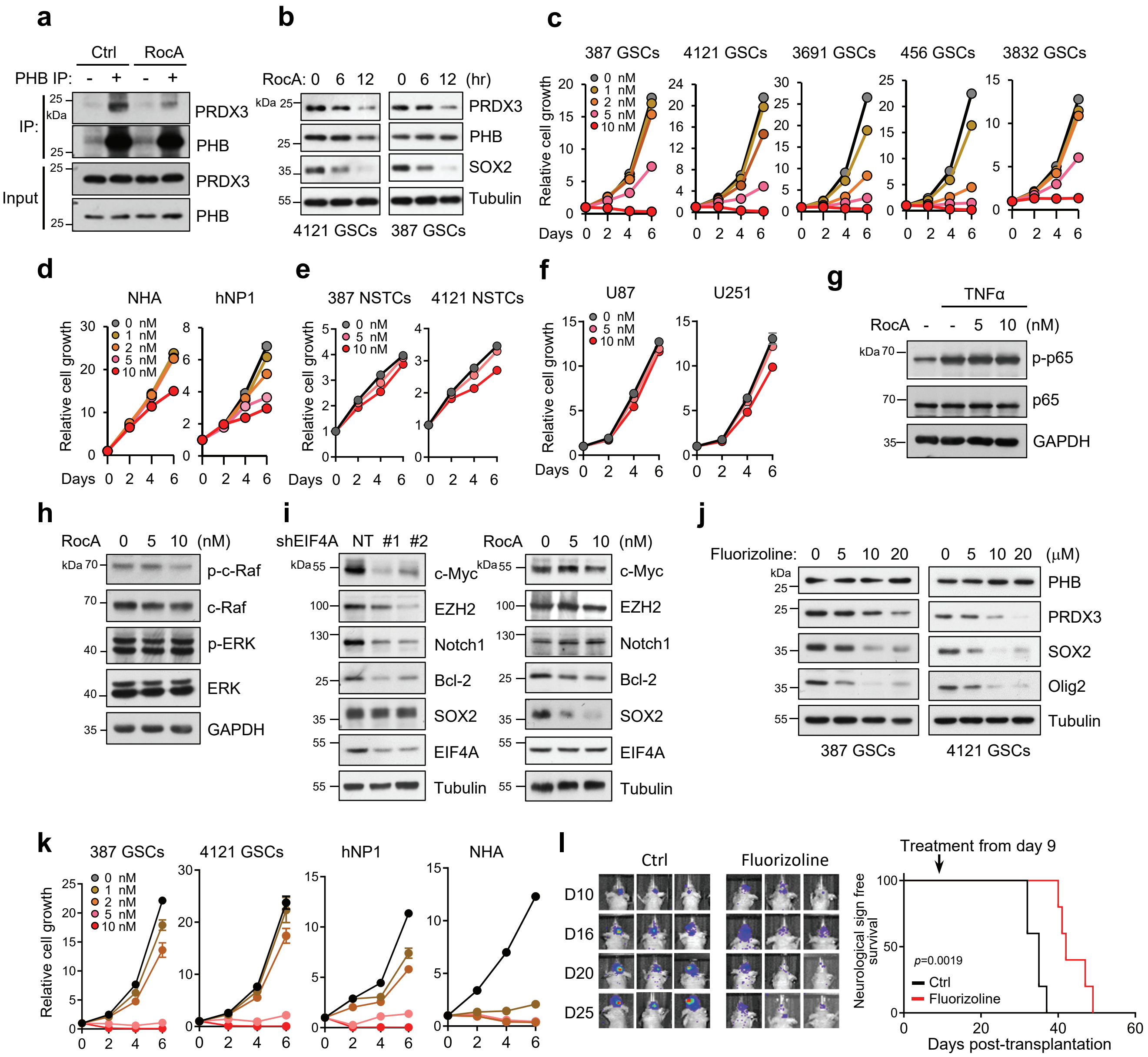

Supplementary Fig. 7 | Pharmacological targeting PHB inhibits GSC growth. a, Co-IP of PHB in 387 GSCs treated with RocA (10nM) and MG132 (10μM) for 12 hours and IB for PHB and PRDX3 are shown. **b,** IB showing the levels of indicated proteins in GSCs treated with RocA (10nM) for indicated time points. **c-f,** Multiple GSC lines (**c**), NHA and hNP1 (**d**), 387 and 4121 NSTCs (**e**), U87 and U251 cells (**f**) were treated with increasing dose of RocA for indicated days, Cell growth were assessed by cell viability assay at indicated time points. (Mean ± SD, n=3, biologically independent experiments). **g-i,** RocA treatment had no obvious effect on phosphorylation of p65 (**g**), activation of c-RAF-ERK signaling pathways (**h**) or EIF4A signaling pathways (**i**) in 4121 GSCs. IB showing the levels of indicated proteins in GSCs treated with increasing dose of RocA for 24 hours. **j,** IB showing the levels of indicated proteins in GSCs treated with Fluorizoline for 12 hours. **k,** GSCs, hNP1 and NHA were treated with increasing dose of Fluorizoline for indicated days. Cell growth were assessed by cell viability assay at indicated time points. (Mean±SD, n=3, biologically independent experiments). **l,** Nude mice (nu/nu) intracranially implanted with 4121 GSCs (Luciferase) were randomly grouped (n=5) at day 9 and treated with or without Fluorizoline (2.5mg/kg, every 3 days). GBM xenografts were tracked by bioluminescence and the representative images are shown (left). Kaplan-Meier survival plot of mice is shown (right, Log rank Mantel-Cox test).

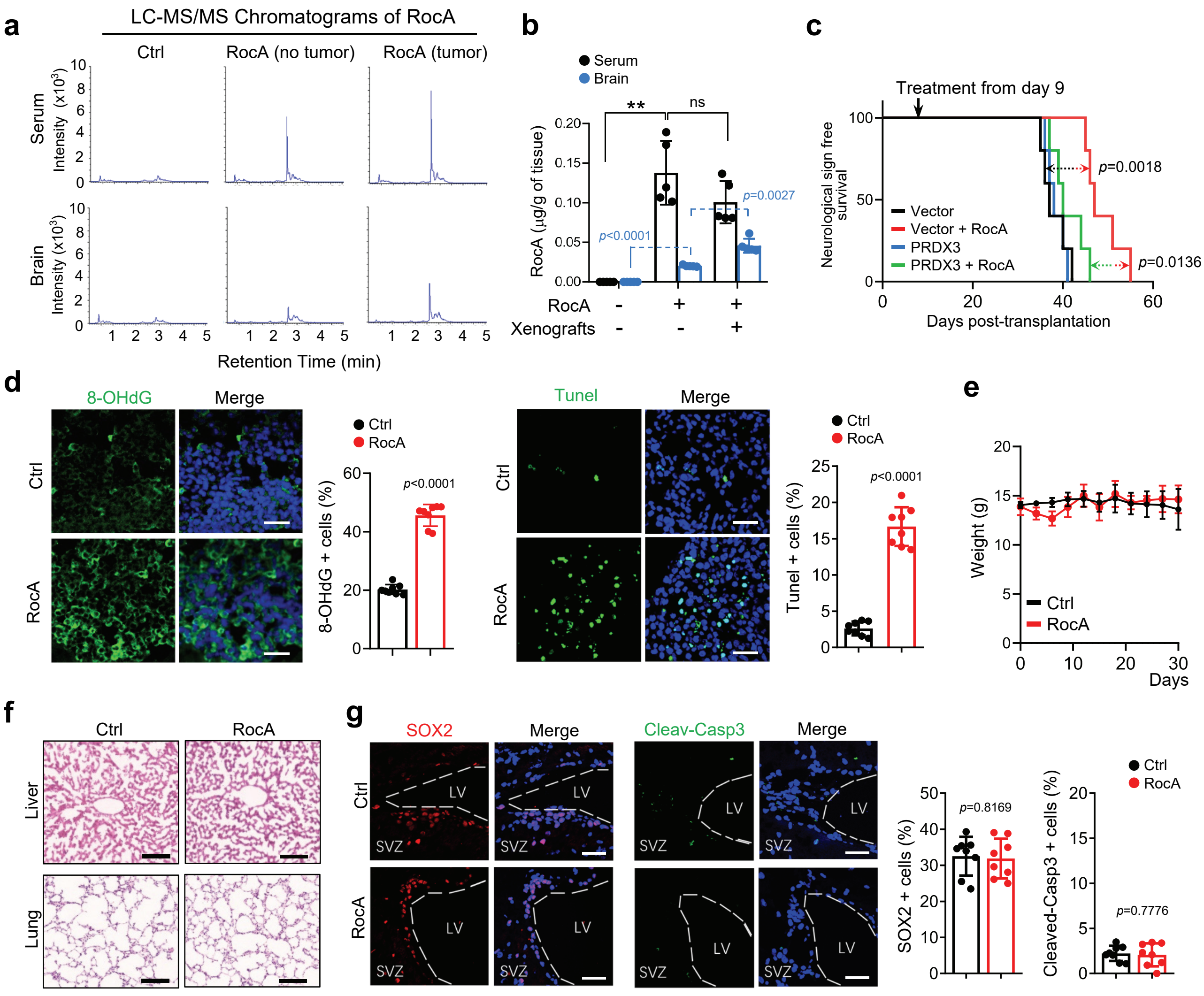

Supplementary Fig. 8 | Pharmacological targeting PHB inhibits GSC-derived tumorigenesis. a,b, Nude mice (nu/nu) with or without GSC-derived orthotopic xenografts (9 days after GSC transplantation) were intraperitoneal injected with RocA (2.5mg/kg). Mice were anaesthetized and blood were collected after RocA treatment for 12 hours. Then, mice were perfused with PBS and sacrificed. Concentration of RocA in serum and brains of mice were measured by LC-MS/MS chromatography. Representative LS-MS/MS chromatograms of RocA in serum and brains of mice are shown (**a**). Quantifications of RocA concentration are shown (**b**). (Mean±SD, n=5, biologically independent samples, Welch's two-sided *t*-test). **c,** Nude mice (nu/nu) intracranially implanted with Flag-vector or Flag-PRDX3 expressed 4121 GSCs were randomly grouped (n=5) at day 9 after implantation and treated with or without RocA (2.5mg/kg, every 3 days, 6 times). Kaplan-Meier survival plot of mice is shown (Log rank Mantel-Cox test). **d,** Nude mice (nu/nu) were treated as described in Fig. 6i. Representative images of IF staining of 8-OHdG or Tunel in GBM xenografts are shown (left). Quantifications of 8-OHdG+ or Tunel+ cells are shown (right). (Mean±SD, images n=8, from 4 biologically independent samples, Welch's two-sided *t*-test). Nuclei were counterstained with Hoechst (blue). Scale bars, 40μm. **e-g,** Nude mice (nu/nu) were treated with control or RocA (2.5mg/kg, every 3 days, 8 times in total). Weight of mice are shown (**e**). (Mean±SD, n=5, biologically independent mice). Representative H&E images of mice liver and lung tissues are shown (**f**). Scale bars, 100μm. Representative IF staining images of SOX2 or Cleaved-caspase 3 in the subventricular zone (SVZ) of mouse brains are shown (**g**, left; Scale bars, 40μm). Quantifications of SOX2+ or Cleaved-caspase 3+ cells are shown (**g**, right). (Mean±SD, images n=8, from 4 biologically independent samples, Unpaired two-sided Student's *t*-test). Nuclei were counterstained with Hoechst (blue).

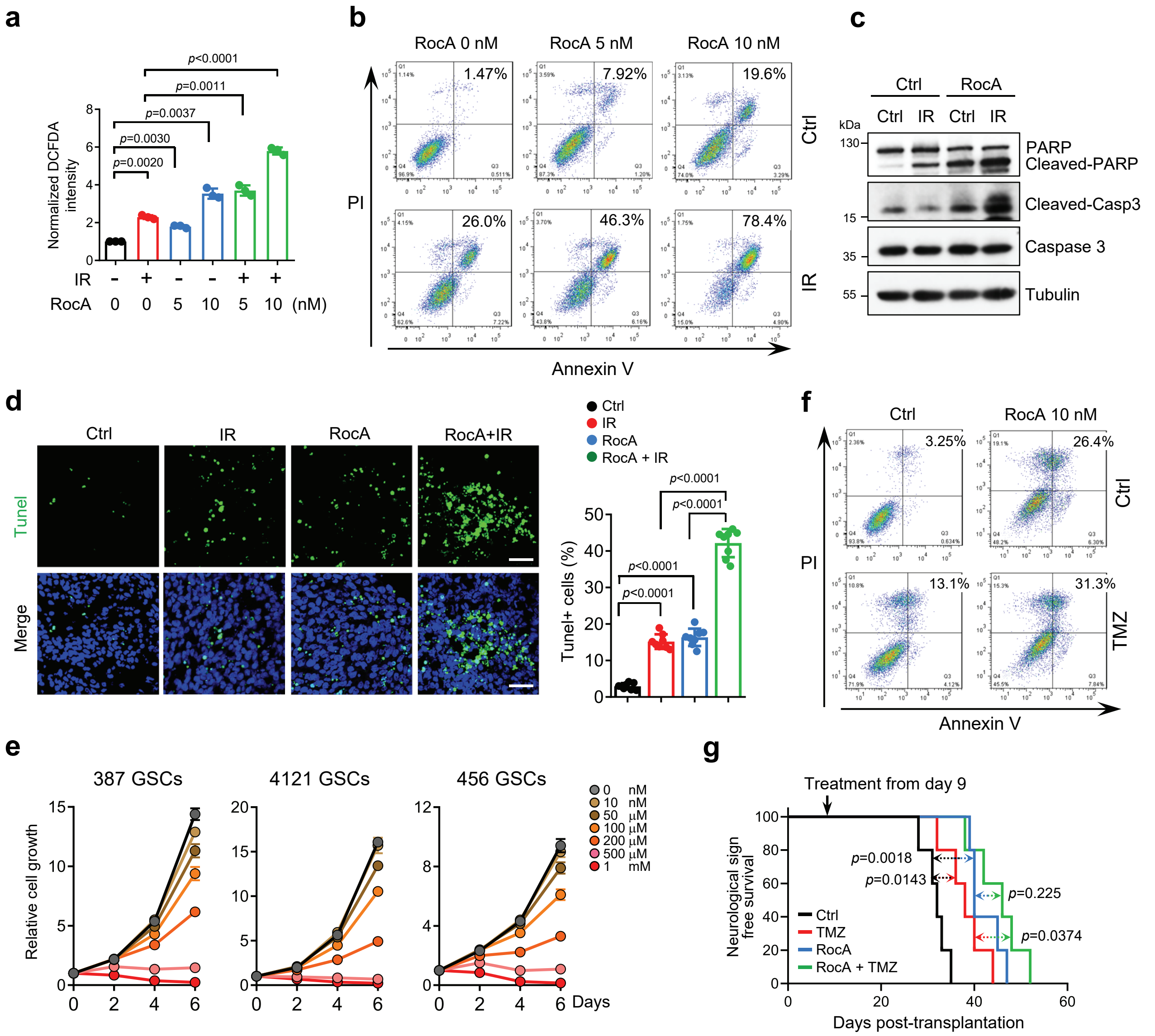

Supplementary Fig. 9 | Pharmacological targeting PHB increases sensitiveness of GSCs to IR. a, The peroxide levels, as indicated by DCFDA fluorescence, were measured by flow cytometry in 387 GSCs with indicated treatments for 48 hours. IR, 3Gy. (Mean±SD, n=3, biologically independent experiments, Welch's two-sided *t*-test). **b,** Cell apoptosis were measured by flow cytometry in 387 GSCs with indicated treatments for 48 hours. IR, 3Gy. **c,** IB showing levels of cleaved-PARP, cleaved-caspaspe3 and caspase3 in 387 GSCs with indicated treatments for 48 hours. IR, 3Gy. **d,** Nude mice (nu/nu) were treated as described in Fig. 7e. IF staining of Tunel in GBM xenografts (4121 GSCs) with indicated treatments. Representative images are shown (left). Quantifications of Tunel+ cells are shown (right). (Mean±SD, images n=8, from 4 biologically independent samples, Welch's two-sided *t*-test). Nuclei were counterstained with Hoechst (blue). Scale bars, 40μm. **e,** GSCs were treated with increasing doses of TMZ. Cell growth were assessed by cell viability assay at indicated time points. (Mean±SD, n=3, biologically independent experiments). **f,** 4121 GSCs were treated with RocA (5nM) and / or TMZ (300 μM) for 48 hours. Cell apoptosis were measured by flow cytometry. **g,** Nude mice (nu/nu) bearing 4121 GSC orthotopic xenografts were randomly grouped (n=5) at day 9 and treated with control, RocA (2.5mg/kg, every 3 days, 6 times), TMZ (60mg/kg, every 3 days, 6 times) or the combination treatment. Kaplan-Meier survival plot of mice are shown (Log rank Mantel-Cox test).

Supplementary Table 1

Expression of PHB and the pathological characteristics of human glioma patients of the Glioma Tissue Microarray.

Tissue identifier	Gender	Age	WHO Grade	Survival condition	Recurrence condition	Disease-free survival (month)	Overall survival (month)	PHB staining intensity	PHB Positive (%)	PHB score
P01A0506	Female	37	II	Live	N	113	113	2	50%	1
P01A0507	Female	20	I	Live	N	113	113	1	30%	0.3
P01A0508	Male	44	I-II	Live	N	113	113	2	80%	1.6
P01A0510	Female	63	III	Live	Recurred	27	112	3	90%	2.7
P01A0509	Male	42	I-II	Live	N	112	112	1	70%	0.7
P01A0663	Male	53	IV	dead	Recurred	22	22	4	70%	2.8
P01A0511	Male	69	I-II	Live	N	111	111	1.5	30%	0.45
P01A0512	Female	11	I	Live	N	111	111	0.5	10%	0.05
P01A0513	Male	16	I-II	Live	N	110	110	1	20%	0.2
P01A0664	Male	24	IV	dead	Recurred	35	52	1	20%	0.2
P01A0515	Male	17	II-III	Live	N	109	109	2	80%	1.6
P01A0516	Female	47	II	Live	N	109	109	1	80%	0.8
P01A0517	Female	34	I	Live	N	108	108	2	60%	1.2
P01A0518	Female	59	II	Live	N	108	108	2	80%	1.6
P01A0519	Female	56	II-III	dead	Recurred	17	72	2	80%	1.6
P01A0521	Male	80	II	Live	Recurred	34	108	2	30%	0.6
P01A0522	Female	28	I-II	Live	N	108	108	1	10%	0.1
P01A0523	Male	62	II-III	Live	N	106	106	0	0	0
P01A0524	Male	66	II	dead	Recurred	83	83	0	0	0
P01A0525	Female	55	I-II	Live	N	106	106	2	30%	0.6
P01A0526	Female	46	I-II	Live	N	106	106	0	0	0
P01A0528	Male	29	III	Live	Recurred	72	105	1	10%	0.1
P01A0529	Female	36	I-II	Live	N	105	105	1	80%	0.8
P01A0530	Female	49	II	Live	Recurred	59	105	2	70%	1.4
P01A0531	Male	61	II	Live	N	105	105	2	30%	0.6
P01A0533	Male	36	II	Live	N	105	105	2	50%	1
P01A0534	Male	39	II-III	Live	Recurred	85	104	2	90%	1.8
P01A0535	Male	56	II	Live	N	104	104	2	30%	0.6
P01A0536	Male	12	I	Live	N	103	103	1	10%	0.1
P01A0537	Male	66	I-II	Live	N	103	103	2	30%	0.6
P01A0538	Female	37	I	Live	N	103	103	1.5	10%	0.15
P01A0539	Male	4	I	Live	N	102	102	1	20%	0.2
P01A0541	Male	6	I-II	Live	N	102	102	2	30%	0.6
P01A0542	Male	5	I	Live	N	102	102	1	50%	0.5
P01A0543	Female	43	II-III	Live	Recurred	62	101	2	80%	1.6
P01A0545	Male	32	I	Live	N	101	101	0	0	0
P01A0544	Male	57	II	Live	N	101	101	3	90%	2.7
P01A0546	Female	55	III	dead	Recurred	81	67	2	20%	0.4
P01A0547	Female	61	I	Live	N	101	101	2	60%	1.2
P01A0548	Male	34	II	Live	N	100	100	2	70%	1.4
P01A0549	Male	37	III	Live	Recurred	46	100	2	50%	1
P01A0550	Male	44	II	Live	Recurred	63	100	1	50%	0.5
P01A0665	Male	37	IV	dead	Recurred	33	33	2	30%	0.6
P01A0552	Male	46	II	Live	N	100	100	1	20%	0.2
P01A0551	Female	52	II	Live	N	99	99	3	80%	2.4
P01A0553	Male	20	I-II	Live	N	99	99	2	10%	0.2
P01A0554	Male	60	II	Live	Recurred	54	99	1	10%	0.1
P01A0555	Female	41	III	dead	Recurred	58	66	2	90%	1.8
P01A0556	Male	51	II	Live	N	99	99	1	60%	0.6
P01A0557	Male	41	II	Live	N	99	99	2	40%	0.8
P01A0558	Male	68	II-III	Live	Recurred	48	99	2	60%	1.2
P01A0559	Male	31	II	Live	Recurred	49	98	2	70%	1.4
P01A0560	Male	63	II	dead	Recurred	55	59	3	70%	2.1
P01A0561	Male	65	I-II	Live	Recurred	34	98	3	50%	1.5

P01A0562	Female	57	II-III	dead	Recurred	50	50	3	70%	2.1
P01A0563	Male	57	II	Live	Recurred	55	97	3	60%	1.8
P01A0687	Female	6	I	Live	N	97	97	1	5%	0.05
P01A0565	Male	47	I-II	Live	Recurred	35	97	3	60%	1.8
P01A0566	Female	42	I-II	Live	N	97	97	1.5	60%	0.9
P01A0568	Male	60	I-II	Live	Recurred	30	97	4	20%	0.8
P01A0569	Male	63	II	Live	Recurred	50	96	4	80%	3.2
P01A0570	Male	20	I-II	Live	N	96	96	0	0	0
P01A0571	Male	69	II	dead	Recurred	36	57	2	50%	1
P01A0572	Female	42	II	Live	Recurred	54	96	1	70%	0.7
P01A0573	Female	26	I	Live	N	95	95	0	0	0
P01A0574	Female	41	III	dead	Recurred	30	30	3	70%	2.1
P02A0003	Male	49	II-III	Live	N	95	95	1	10%	0.1
P01A0666	Male	41	IV	dead	Recurred	41	50	2	90%	1.8
P01A0667	Male	44	IV	dead	Recurred	13	13	3	90%	2.7
P01A0575	Female	36	II	Live	N	94	94	3	60%	1.8
P01A0668	Male	22	IV	dead	Recurred	11	11	4	90%	3.6
P01A0576	Male	20	II-III	Live	Recurred	27	94	1	40%	0.4
P01A0577	Male	36	I-II	Live	N	93	93	1	50%	0.5
P01A0579	Female	18	I-II	Live	N	93	93	3	30%	0.9
P01A0580	Female	42	II-III	Live	N	93	93	1	60%	0.6
P01A0581	Male	41	II	dead	Recurred	47	55	3	80%	2.4
P01A0582	Male	58	II-III	dead	Recurred	14	73	2	70%	1.4
P01A0583	Male	37	II-III	dead	Recurred	56	56	2	60%	1.2
P01A0585	Male	35	II-III	Live	N	92	92	2	50%	1
P01A0669	Female	43	IV	dead	Recurred	12	15	3	80%	2.4
P01A0586	Male	40	I-II	Live	Recurred	26	92	1	10%	0.1
P01A0587	Male	46	II-III	dead	Recurred	27	37	1	50%	0.5
P01A0588	Female	17	I-II	Live	N	91	91	1	30%	0.3
P01A0589	Female	47	II-III	Live	Recurred	43	91	2	70%	1.4
P01A0590	Male	33	II-III	Live	N	91	91	0	0	0
P01A0594	Male	79	I-II	dead	Recurred	33	33	3	90%	2.7
P01A0595	Male	74	II	dead	Recurred	20	20	3	100%	3
P01A0596	Male	50	I-II	Live	N	88	88	1	10%	0.1
P01A0597	Female	36	I-II	Live	N	88	88	1	50%	0.5
P01A0598	Male	32	II	Live	Recurred	41	88	1	60%	0.6
P01A0599	Male	36	III	Live	Recurred	30	88	3	80%	2.4
P01A0600	Male	38	I	Live	N	88	88	1	20%	0.2
P01A0601	Female	18	I	Live	N	87	87	1	30%	0.3
P01A0602	Male	3	I-II	Live	N	87	87	1	5%	0.05
P01A0603	Female	42	II	Live	Recurred	53	87	2	80%	1.6
P01A0604	Female	45	II-III	dead	Recurred	35	55	2	90%	1.8
P01A0607	Male	68	II	dead	Recurred	44	62	3	60%	1.8
P01A0670	Male	60	IV	dead	Recurred	10	10	4	80%	3.2
P01A0608	Female	33	III	dead	Recurred	33	33	1	60%	0.6
P01A0671	Female	65	IV	dead	Recurred	23	23	3	80%	2.4
P01A0609	Female	14	I	Live	N	86	86	1	50%	0.5
P01A0610	Male	28	I-II	Live	N	86	86	2	50%	1
P01A0611	Male	37	I-II	Live	N	86	86	2	90%	1.8
P01A0672	Female	60	IV	dead	Recurred	17	17	3	80%	2.4
P01A0612	Female		I-II	Live	N	85	85	3	70%	2.1
P01A0613	Male	36	II	Live	N	85	85	1	40%	0.4
P01A0614	Female	49	I	Live	N	85	85	1	50%	0.5
P01A0615	Male	41	III	dead	Recurred	32	32	3	80%	2.4
P01A0616	Male	19	II	Live	N	84	84	1	60%	0.6
P01A0617	Male	59	III	dead	Recurred	11	11	2	85%	1.7
P01A0618	Male	41	I-II	Live	N	84	84	2	50%	1
P01A0673	Male	56	IV	dead	Recurred	32	32	4	90%	3.6
P01A0619	Male	58	II-III	dead	Recurred	40	40	1	5%	0.05
P01A0620	Female	40	III	dead	Recurred	26	27	1	10%	0.1
P01A0621	Male	33	III	Live	N	82	82	0	0	0

P01A0622	Male	35	II-III	dead	Recurred	22	59	2	70%	1.4
P01A0623	Female	38	I	Live	N	81	81	1	10%	0.1
P01A0624	Male	47	III	Live	Recurred	33	81	3	60%	1.8
P01A0625	Male	34	II-III	Live	Recurred	40	81	3	50%	1.5
P01A0674	Male	46	IV	dead	Recurred	34	41	3	50%	1.5
P01A0628	Female	58	II-III	dead	Recurred	34	34	3	20%	0.6
P02A0004	Female	59	II-III	Live	N	79	79	3	50%	1.5
P01A0629	Female	30	III	dead	Recurred	23	23	2	70%	1.4
P01A0676	Male	65	IV	dead	Recurred	23	34	3	90%	2.7
P01A0677	Male	9	IV	dead	Recurred	17	17	0	0	0
P01A0678	Male	41	IV	dead	Recurred	12	12	2	90%	1.8
P01A0630	Male	49	III	dead	Recurred	26	26	2	90%	1.8
P01A0680	Male	78	IV	dead	Recurred	6	6	3	70%	2.1
P01A0631	Female	48	II	Live	Recurred	26	77	1	80%	0.8
P01A0633	Male	36	II	Live	Recurred	41	77	0	0	0
P01A0634	Male	40	I-II	Live	N	77	77	1	10%	0.1
P01A0635	Male	41	II	Live	Recurred	76	76	0	0	0
P01A0636	Female	45	II	Live	N	76	76	1	5%	0.05
P01A0637	Male	54	I-II	Live	N	76	76	1	20%	0.2
P01A0638	Female	32	II	Live	N	76	76	0	0	0
P01A0639	Male	48	II	Live	N	75	75	1	20%	0.2
P01A0641	Female	24	I-II	Live	N	75	75	2	20%	0.4
P01A0642	Male	52	II-III	dead	Recurred	23	23	3	70%	2.1
P01A0643	Female	26	II-III	Live	Recurred	75	75	4	60%	2.4
P01A0681	Male	62	IV	dead	Recurred	10	10	3	80%	2.4
P01A0644	Male	56	II	Live	N	74	74	1	90%	0.9
P01A0645	Male	15	II	Live	N	74	74	2	60%	1.2
P01A0682	Male	67	III	dead	Recurred	19	19	3	90%	2.7
P01A0646	Male	30	I	Live	N	72	72	2	50%	1
P01A0647	Female	70	II-III	dead	Recurred	29	29	3	70%	2.1
P01A0649	Male	41	II	dead	Recurred	26	26	0	0	0
P01A0683	Male	63	IV	dead	Recurred	9	9	1	20%	0.2
P01A0684	Male	45	IV	dead	Recurred	10	10	4	80%	3.2
P01A0650	Male	37	II	Live	N	71	71	0	0	0
P01A0651	Female	69	III	dead	Recurred	11	11	1	70%	0.7
P01A0653	Female	19	II	Live	N	71	71	1	50%	0.5
P01A0652	Male	49	II	Live	N	71	71	1	40%	0.4
P01A0654	Female	66	I	Live	N	71	71	1	10%	0.1
P01A0685	Male	41	IV	dead	Recurred	14	14	2	70%	1.4
P01A0655	Male	56	III	dead	Recurred	21	21	4	60%	2.4
P01A0656	Female	20	I	Live	N	70	70	1	20%	0.2
P01A0658	Male	57	III	dead	Recurred	37	37	2	40%	0.8
P01A0659	Male	10	I	Live	N	69	69	1	10%	0.1
P01A0660	Female	36	II-III	Live	Recurred	69	69	2	80%	1.6
P01A0661	Male	40	II-III	dead	Recurred	39	39	3	90%	2.7

Supplementary Table 2

The sequences of the sgRNAs and shRNAs

PHB-sgRNA#1	5'- AGGATCTCAGTTGTGATGGA-3'
PHB-sgRNA#2	5'-GACTCATTTTCTCATCCCGT-3'
PHB DOX-induced shRNA	5'- GAGTTCACAGAAGCGGTGGAA -3'
PHB-shRNA#1	5'-CCCAGAAATCACTGTGAAATT-3'
PHB-shRNA#2	5'- GAGTTCACAGAAGCGGTGGAA-3'
PRDX3-shRNA#1	5'-CCAGATGGTCAGTTTAAAGAT-3'
PRDX3-shRNA#2	5'-GATGAGACTTTGAGACTAGTT-3'
eIF4A1-shRNA#1	5'-GCCGTAAAGGTGTGGCTATTA-3'
eIF4A1-shRNA#2	5'-CCTTGTATCAAGGGTTATGAT-3'

Supplementary Table 3

Primer sequences used for qRT-PCR analysis

UNC5B	Forward 5'- GTCGGACACTGCCAACTATAC -3'
	Reverse 5'- CCGCCATTCACGTAGACGAT -3'
DDIT3	Forward 5'- GGAAACAGAGTGGTCATTCCC -3'
	Reverse 5'- CTGCTTGAGCCGTTCATTCTC -3'
TRIB3	Forward 5'- AAGCGGTTGGAGTTGGATGAC -3'
	Reverse 5'- CACGATCTGGAGCAGTAGGTG -3'
ATF3	Forward 5'- CCTCTGCGCTGGAATCAGTC -3'
	Reverse 5'- TTCTTTCTCGTCGCCTCTTTTT -3'
NUPR1	Forward 5'- CTCTCATCATGCCTATGCCTACT -3'
	Reverse 5'- CCTCCACCTCCTGTAACCAAG -3'
ALDH1L2	Forward 5'- GCTGAAGTTGGCACTAATTGGC -3'
	Reverse 5'- TGAACACCCCTACTACTCGGT -3'
INHBE	Forward 5'- ATCTTCCGATGGGGACCAAG -3'
	Reverse 5'- AGAGTTAAGGTATGCCAGCCC -3'
DDR2	Forward 5'- GCTATATGCCGCTATCCTCTGG -3'
	Reverse 5'- ACTCTGACCACTGACTGGAAG -3'
PHB	Forward 5'- TGTCATCTTTGACCGATTCCG -3'
	Reverse 5'- CTGGCACATTACGTGGTCGAG -3'
PRDX3	Forward 5'- ACAGCCGTTGTCAATGGAGAG -3'
	Reverse 5'- ACGTCGTGAAATTCGTTAGCTT -3'
β-Actin	Forward 5'- AGAAAATCTGGCACCACACC -3'
	Reverse 5'- AGAGGCGTACAGGGATAGCA -3'
GAPDH	Forward 5'- CCAGGTGGTCTCCTCTGACTTC -3'
	Reverse 5'- GTGGTCGTTGAGGGCAATG -3'
Bulge-Loop hsa-miR-27a-3p Primer Set	RiboBio, MQPS0000891-1-100
Bulge-Loop U6 qPCR Primer Set	RiboBio, MQPS0000002-1-100
micrON mimic NC #22	RiboBio, miR1N0000001-1-5
micrON hsa-miR-27a-3p mimic	RiboBio, miR10000084-1-5
micrOFF inhibitor NC #22	RiboBio, miR2N0000001-1-5
micrOFF hsa-miR-27a-3p inhibitor	RiboBio, miR20000084-1-5
