# Supplementary material for: Suppression of mitochondrial ROS by prohibitin drives glioblastoma progression and therapeutic resistance
Source: Nat Commun. 2021 Jun 17;12:3720. doi: 10.1038/s41467-021-24108-6 (PMC8211793; doi:10.1038/s41467-021-24108-6)
Supplement: Supplementary file 2 — Reporting Summary [file 41467_2021_24108_MOESM2_ESM.pdf]

nature research

Corresponding author(s): Jianghong Man

Last updated by author(s): May 20, 2021

Reporting Summary

Nature Research wishes to improve the reproducibility of the work that we publish. This form provides structure for consistency and transparency in reporting. For further information on Nature Research policies, see our Editorial Policies and the Editorial Policy Checklist.

Statistics

For all statistical analyses, confirm that the following items are present in the figure legend, table legend, main text, or Methods section.

n/a	Confirmed	
☐	☒	The exact sample size (*n*) for each experimental group/condition, given as a discrete number and unit of measurement
☐	☒	A statement on whether measurements were taken from distinct samples or whether the same sample was measured repeatedly
☐	☒	The statistical test(s) used AND whether they are one- or two-sided<br>*Only common tests should be described solely by name; describe more complex techniques in the Methods section.*
☒	☐	A description of all covariates tested
☐	☒	A description of any assumptions or corrections, such as tests of normality and adjustment for multiple comparisons
☐	☒	A full description of the statistical parameters including central tendency (e.g. means) or other basic estimates (e.g. regression coefficient) AND variation (e.g. standard deviation) or associated estimates of uncertainty (e.g. confidence intervals)
☐	☒	For null hypothesis testing, the test statistic (e.g. *F*, *t*, *r*) with confidence intervals, effect sizes, degrees of freedom and *P* value noted<br>*Give P values as exact values whenever suitable.*
☒	☐	For Bayesian analysis, information on the choice of priors and Markov chain Monte Carlo settings
☒	☐	For hierarchical and complex designs, identification of the appropriate level for tests and full reporting of outcomes
☐	☒	Estimates of effect sizes (e.g. Cohen's *d*, Pearson's *r*), indicating how they were calculated

Our web collection on statistics for biologists contains articles on many of the points above.

Software and code

Policy information about availability of computer code

Data collection	Xenograft growth was monitored by bioluminescent imaging using the Calibar IVIS® Spectrum (PerkinElmer). Images were collected using a Zeiss LSM880 system. qPCR was performed using StepOnePlus (Applied Biosystems). Flow cytometry was performed using BD FACS Aria III. RNA sequencing were performed using the Agilent 2100 BioAnalyzer (Agilent Technologies). RocA were analyzed by Triple Quad 6500 mass spectrometer (SCIEX) coupled with the UltiMate 3000 HPLC system (Thermo Fisher Scientific).
Data analysis	For the liquid chromatography-tandem mass spectrometry analysis of rocaglamide, Peak integration and statistical analyses were performed using MultiquantTM 2.1 software (SCIEX). The acquisition software for immunofluorescent images was Zen 2.1 SP2. Fluorescent intensity was analyzed using the ImageJ 1.8.0 software (NIH). Flow cytometry was analyzed using the FlowJo 7.6 software. Extremely limiting dilution analysis was performed using software available at http://bioinf.wehi.edu.au/software/elda. For RNA sequencing, reads were aligned to the GRCh38.p7 genome using TopHat v2.1.1 with the library type option set to first strand. Fragments Per Kilobase per Millions (FPKMs) of known genes were calculated using eXpress v1.5.1. Statistical analyses were performed using GraphPad Prism 8.0 and Microsoft Office Excel (office 2013).

For manuscripts utilizing custom algorithms or software that are central to the research but not yet described in published literature, software must be made available to editors and reviewers. We strongly encourage code deposition in a community repository (e.g. GitHub). See the Nature Research guidelines for submitting code & software for further information.

Data

Policy information about availability of data

All manuscripts must include a data availability statement. This statement should provide the following information, where applicable:

- Accession codes, unique identifiers, or web links for publicly available datasets
- A list of figures that have associated raw data
- A description of any restrictions on data availability

The microarray data referenced during the study are available from the Gene Expression Omnibus under accession number GSE86237 and GSE54791. The expression of PHB and the pathological characteristics of human glioma patients of the tissue microarray are provided in Supplementary Table 1. The different expressed genes in control and PHB KO GSCs are provided in Supplementary Table 2. The primer sequences used for Q-PCR are provided in Supplementary Table 3. Source data are provided with this paper. All data supporting the findings of this study are available from the corresponding authors upon reasonable request.

Field-specific reporting

Please select the one below that is the best fit for your research. If you are not sure, read the appropriate sections before making your selection.

[x] Life sciences   [ ] Behavioural & social sciences   [ ] Ecological, evolutionary & environmental sciences

For a reference copy of the document with all sections, see nature.com/documents/nr-reporting-summary-flat.pdf

Life sciences study design

All studies must disclose on these points even when the disclosure is negative.

Sample size	No statistic method was used to predetermine sample size. The sample sizes were determined based on previous experience with similar experimental systems (Jianghong Man et al., Cell Stem Cell, 2018, Jan 4;22(1):104-118.e6). We usually use at least 5 mice per group. The sample size is sufficient for a confident data analysis and in our experience is sufficient to control for technical variations. The number of the independent experiments was indicated in each figure legend.
Data exclusions	No data were excluded from the analyses.
Replication	Every experiments were independently repeated at least three times with similar results. Some results were confirmed across multiple cell lines and tumor samples with independent experiments. All replication attempts were successful.
Randomization	For in vitro assays, cells were randomly assigned to either experiment or control group. A biological sample was split into the equal parts for control and experiment groups. For tumor initiation experiments, mice were randomly divided into control and experimental groups at the start of each experiment. For the combinational therapy, the tumor bearing mice were grouped randomly when tumors reached a similar size, and were treated as indicated in the paper.
Blinding	For all the experiments including imaging, expression correlations and survival analyses, the investigators were blinded to group allocation during data analysis. The investigators were not blinded during data collection for the in vivo and in vitro experiments. Blinding was not applicable for in vivo experiments as mice were receiving different treatments.

Reporting for specific materials, systems and methods

We require information from authors about some types of materials, experimental systems and methods used in many studies. Here, indicate whether each material, system or method listed is relevant to your study. If you are not sure if a list item applies to your research, read the appropriate section before selecting a response.

Materials & experimental systems

n/a	Involved in the study	
[ ]	[x]	Antibodies
[ ]	[x]	Eukaryotic cell lines
[x]	[ ]	Palaeontology and archaeology
[ ]	[x]	Animals and other organisms
[ ]	[x]	Human research participants
[x]	[ ]	Clinical data
[x]	[ ]	Dual use research of concern

Methods

n/a	Involved in the study	
[x]	[ ]	ChIP-seq
[ ]	[x]	Flow cytometry
[x]	[ ]	MRI-based neuroimaging

Antibodies

Antibodies used	Anti-PHB (ab75766, Lot: GR24874-19, clone number: EP2803Y), Abcam, dilution 1/5000 for IB, 1/200 for IF, 1/100 for IHC;<br>Anti-SOX2 (MAB4423, Lot: 2999814, clone number:10H9.1), Millipore, dilution 1/1000 for IB;

Anti-SOX2 (sc-365823, Lot: #G0717, clone number: E-4), Santa Cruz, dilution, 1/200 for IF;
Anti-Olig2 (sc-48817, Lot: #C1413), Santa Cruz, dilution 1/1000 for IB, 1/200 for IF;
GFAP (12389s, Lot: 5, clone number: D1F4Q), Cell Signaling Technology, dilution1/1000 for IB;
PRDX3 (NBD2-67043, Lot: HK0703, clone number: JA53-21) Novus, for IB, 1:5000; for IF, 1:200)
8-OHdG (ab48508, Lot: GR3173165-5), Abcam, dilution 1/400 for IF;
TOM20 (sc-17764, Lot: # K0314, clone number:H-8), Santa Cruz, dilution 1/400 for IF;
TIM23 (sc514463, Lot: #F2016, clone number: F-10), Santa Cruz, dilution 1/200 for IF;
Tubulin (T5168, Lot: #103M4773V, clone number: B-5-1-2) Sigma, dilution 1/5000 for IB;
Caspase3 (19677-1-AP) Proteintech, dilution 1/1000for IB;
Cleaved-Caspase3 (9661s, Lot: 45, clone number: Asp175), Cell Signaling Technology, dilution 1/1000 for IB, 1/100 for IF;
PARP (9542, Lot: 15), Cell Signaling Technology, dilution 1/1000 for IB; Flag (F3165), Sigma, dilution 1/2000 for IB;
PRDX1 (158-1-AP, Lot: 00023079), Proteintech, dilution 1/1000 for IB;
PRDX6 (13585-1-AP, Lot: 00046546), Proteintech, dilution 1/1000 for IB;
GPX1 (3206s, Lot: 2), Cell Signaling Technology, dilution 1/1000 for IB;
Mn-SOD (66474-l-lg, Lot: 10004881, clone number:3A6C2), Proteintech, dilution 1/1000 for IB;
PHB2 (14085s, Lot: 1, clone number: E1Z5A), Cell Signaling Technology, dilution 1/1000 for IB;
β-Actin (sc-47778, Lot: #G0213, clone number: C4), Santa Cruz, dilution 1/1000 for IB;
Ubiquitin (D058-3, Lot: 045), MBL, dilution 1/1000 for IB;
Ubiquitin, Lys48-specific (05-1307, Lot: 3241199, clone number: Apu2), Millipore, dilution 1/1000 for IB;
p65 (sc-8008, Lot:B1218, clone number:F-6), Santa Cruz, dilution 1/500 for IB;
phospho-p65 (3033s, Lot:17, clone number:93H1), Cell Signaling Technology, dilution 1/1000 for IB;
c-Raf (12552s, Lot:1, clone number: D5X6R), Cell Signaling Technology, dilution 1/1000 for IB;
Phospho-c-Raf (9427s, Lot: 10, clone number: s338) Cell Signaling Technology, dilution 1/1000 for IB;
P44/42 MAPK (ERK1/2) (4695s, Lot: 28, clone number:13TF5) Cell Signaling Technology, dilution 1/500 for IB;
Phospho-p44/42 MAPK (Erk1/2) (4370s, Lot: 24, clone number: D13.14.4E), Cell Signaling Technologies, dolition 1/500 for IB;
eIF4A1 (2490T, Lot: 2), Cell Signaling Technology, dilution 1/1000for IB;
Notch1 (3608p, Lot: 3, clone number: D1E11), Cell Signaling Technology, dilution 1/1000for IB;
c-Myc (sc-40, Lot#L1318, clone number:9E10), Santa Cruz, Cell Signaling Technology, dilution 1/1000 for IB;
Bcl-2 (sc-7382, Lot#A1719, clone number:C-2), Santa Cruz, dilution 1/500 for IB;
Ezh2 (5246T, Lot: 9, clone number: D2C9), Cell Signaling Technology, dilution 1/1000 for IB;
Cu-ZnSOD (10269-1-AP, Lot: 00091172), Proteintech, dilution 1/1000 for IB;
γH2AX (05-636, Lot: 3292608, clone number: JBW301), Millipore, dilution 1/1000 for IB.

Secondary antibody labeled with polymer-HRP (horseradish peroxidase) anti-rabbit
(111-035-003, Lot： 147832), Jackson, dilution 1/5000 for IB
Secondary antibody labeled with polymer-HRP (horseradish peroxidase) anti-mouse
(115-035-003, Lot： 148148), Jackson, dilution 1/5000 for IB
Alexa Fluor 488 donkey anti-rabbit IgG(H+L) (A21206, Lot:1927937), Thermo, dilution 1/400 for IF
Alexa Fluor 488 donkey anti-mouse IgG(H+L) (A21202, Lot:1915874), Thermo, dilution 1/400 for IF
Alexa Fluor 555 donkey anti-mouse IgG(H+L) (A31570, Lot:1905844), Thermo, dilution 1/400 for IF
Alexa Fluor 555 donkey anti-goat IgG(H+L) (A21432, Lot:1932497), Thermo, dilution 1/400 for IF

Validation

All the antibodies used are commercially available. Antibody validation was provided by the company. Representative western blot is shown for a number of antibodies within Figures 1, 2, 3, 4, 5 ,6 ,7 and Supplementary Figures 1, 2, 3, 4 ,5, 6 ,7 ,9. Representative staining is shown for a number of antibodies within Figures 1, 2, 3, 5 ,6 and Supplementary Figures 1, 3 ,4 ,5, 8 ,9. Information of antibody validation is provided on manufacturer's official website, which shows validated data from multiple species of cells for the experiment of WB, IF, IP, IHC and so on. Validation data can be accessed by searching for the listed catalog numbers on the Abcam (https://www.abcam.cn/), Millipore (https://www.merckmillipore.com/CN/zh), Santa Cruz (https://www.scbt.com/zh/home), Cell Signaling Technology (https://www.cellsignal.cn/), Novus (https://www.novusbio.com/), Sigma (https://www.sigmaaldrich.cn/CN/zh), Proteintech (https://www.ptgcn.com/), MBL (http://www.mbl-chinawide.cn/), Jackson (https://www.jacksonimmuno.com/) or Thermo (https://www.thermofisher.com/) websites.

Eukaryotic cell lines

Policy information about cell lines

Cell line source(s)

GSCs (387, 3691, 3832, 3359, 3264, 4121, 456) were a gift from Dr. Jeremy Rich (University of California, San Diego) and Jennifer Yu (Cleveland Clinic). NPCs (hNP1, 15167, 17231) were a gift from Dr. Shideng Bao (Cleveland Clinic). U87, U251, HEK293 were purchased from the American Type Culture Collection. NHA was purchased from Beina Chuanglian Biotechnology Institute.

Authentication

The GSCs (387, 3691, 3832, 3359, 3264, 4121, 456) and NPCs (hNP1, 15167, 17231) have been validated by the supplier through genetic profiling and functional validation. U87 (from ATCC) , U251 (from ATCC) and NHA (from Beina Chuanglian Biotechnology Institute) were authenticated by the organizations with certificates.

Mycoplasma contamination

All cell lines were tested and free of mycoplasma contamination.

Commonly misidentified lines
(See ICLAC register)

No commonly misidentified cell lines were used.

Animals and other organisms

Policy information about studies involving animals; ARRIVE guidelines recommended for reporting animal research

Laboratory animals	Mice used in our studies were 4-wk-old female NU/NU nude mice purchased from Beijing Vital River Laboratory Animal Technology. Patient-derived xenografts (PDXs) were established through subcutaneous transplantation of GBM patient cells into NOD/SCID mice. All NOD/SCID mice were 4-week-old females. Mice were kept under specific-pathogen-free conditions, with controlled temperature (20-25℃), humidity (40-60%) and light cycle (12 h light/dark).
Wild animals	No wild animals were used.
Field-collected samples	No field-collected samples were used.
Ethics oversight	All animal experiments were performed in accordance with the NIH guide for the care and use of laboratory animals and with the approval of the Institutional Animal Care and Use Committee of National Center of Biomedical Analysis.

Note that full information on the approval of the study protocol must also be provided in the manuscript.

Human research participants

Policy information about studies involving human research participants

Population characteristics	*Describe the covariate-relevant population characteristics of the human research participants (e.g. age, gender, genotypic information, past and current diagnosis and treatment categories). If you filled out the behavioural & social sciences study design questions and have nothing to add here, write "See above."*
Recruitment	*Describe how participants were recruited. Outline any potential self-selection bias or other biases that may be present and how these are likely to impact results.*
Ethics oversight	*Identify the organization(s) that approved the study protocol.*

Note that full information on the approval of the study protocol must also be provided in the manuscript.

Flow Cytometry

Plots

Confirm that:

- [x] The axis labels state the marker and fluorochrome used (e.g. CD4-FITC).
- [x] The axis scales are clearly visible. Include numbers along axes only for bottom left plot of group (a 'group' is an analysis of identical markers).
- [x] All plots are contour plots with outliers or pseudocolor plots.
- [x] A numerical value for number of cells or percentage (with statistics) is provided.

Methodology

Sample preparation	Cells were harvested and trypsinized as single cell, washed with PBS, re-suspended in 10 μM CM-H2DCFDA (Thermo Fisher Scientific, c6827) in PBS and stained for 15 minutes at 37°C in the incubator. After staining, cells were washed twice with PBS and then re-suspended in PBS. H2DCFDA fluoresce signaling was detected by flow cytometer at 488 nm channel, and totally 10,000 cells were analyzed per sample. Data analysis was performed with FlowJo 7.6 software.
Instrument	BD FACS Aria III
Software	FlowJo 7.6 software.
Cell population abundance	In each experiment the same number of cells were recorded for every condition.
Gating strategy	Gates and regions are placed around populations of cells with defined characteristics.

- [x] Tick this box to confirm that a figure exemplifying the gating strategy is provided in the Supplementary Information.
